# Transcriptomic and metabolomic analyses reveal the potential mechanism of waterlogging resistance in cotton (*Gossypium hirsutum* L.)

**DOI:** 10.3389/fpls.2023.1088537

**Published:** 2023-06-12

**Authors:** Anane Gideon Owusu, Yin-Ping Lv, Man Liu, Yong Wu, Cheng-Lin Li, Ning Guo, Da-Hui Li, Jun-Shan Gao

**Affiliations:** School of Life Sciences, Anhui Agricultural University, Hefei, China

**Keywords:** cotton, waterlogging stress, transcriptomics, metabolomics, differentially expressed genes, transcription factors

## Abstract

**Introduction:**

Cotton (*Gossypium hirsutum* L.) is susceptible to long-term waterlogging stress; however, genomic information of cotton response mechanisms toward long days of waterlogging is quite elusive.

**Methods:**

Here, we combined the transcriptome and metabolome expression level changes in cotton roots after 10 and 20 days of waterlogging stress treatment pertaining to potential resistance mechanisms in two cotton genotypes.

**Results and discussion:**

Numerous adventitious roots and hypertrophic lenticels were induced in CJ1831056 and CJ1831072. Transcriptome analysis revealed 101,599 differentially expressed genes in cotton roots with higher gene expression after 20 days of stress. Reactive oxygen species (ROS) generating genes, antioxidant enzyme genes, and transcription factor genes (*AP2*, *MYB*, *WRKY*, and *bZIP*) were highly responsive to waterlogging stress among the two genotypes. Metabolomics results showed higher expressions of stress-resistant metabolites sinapyl alcohol, L-glutamic acid, galactaric acid, glucose 1-phosphate, L-valine, L-asparagine, and melibiose in CJ1831056 than CJ1831072. Differentially expressed metabolites (adenosine, galactaric acid, sinapyl alcohol, L-valine, L-asparagine, and melibiose) significantly correlated with the differentially expressed *PRX52*, *PER1*, *PER64*, and *BGLU11* transcripts. This investigation reveals genes for targeted genetic engineering to improve waterlogging stress resistance to enhance abiotic stress regulatory mechanisms in cotton at the transcript and metabolic levels of study.

## Introduction

Waterlogging is a major type of flooding problem, depending on the water level encountered by plant species (Jia et al., 2021). It primarily causes oxygen deficiency in soil in plant roots, resulting in morphological, physiological, molecular, and anatomical changes in plant tissues that affect plant growth and development. Typically, major damage from waterlogging stress is similar but species-specific. However, very common intrinsic disruptive substances and reactive oxygen species, such as hydrogen peroxides (H_2_O_2_) (Xu et al., 2013) and superoxide anion radical ( ${\text{O}}_{2}^{\cdot -}$ ) (Wang et al., 2018a) and malondialdehyde (MDA) (Tian et al., 2019), usually accumulate under oxidative conditions posed by waterlogging. These substances are detrimental to plant growth and development because they reduce plant photosynthesis by causing leaf chlorosis, root death, and a reduction in chlorophyll content and chloroplast damage (Parent et al., 2008; Broughton et al., 2015; Zhang et al., 2022). In contrast, when these substances are activated during waterlogging, plants also use defensive mechanisms and counteract them by adapting to anaerobic conditions. Adaptive mechanisms are normally characterized morphologically, biochemical, anatomically, and molecularly, with the aim of discovering resistance mechanisms for plant survival. Popularly reported morphological defensive mechanisms mainly include adventitious root formation (Broughton et al., 2015), hypertrophic lenticel formation (Le Provost et al., 2016), aerenchyma air space formation (Chávez-Arias et al., 2019), radial oxygen loss spaces (Dodd et al., 2013), and formation of apical meristem (Blokhina et al., 2001). Biochemical defensive mechanisms consist of the production of enzymatic scavengers such as peroxidase (POD), superoxide dismutase (SOD), dehydroascorbate reductase (DHAR), catalase (CAT), monodehydroascorbate reductase, (MDHAR), glutathione reductase (GR), and non-enzymatic scavengers, such as proline (Zhang et al., 2016; Chávez-Arias et al., 2019; Hasanuzzaman et al., 2020; Park et al., 2020). In addition, hormone biosynthesis, such as ethylene abscisic acid, gibberellins, and auxin biosynthesis, has been reported to help in plant signaling and resistance to waterlogging stress (Coutinho et al., 2018; Zhao et al., 2018).

Cotton is a prevalent crop due to its chief source of high-quality fiber, making it an important asset to mankind. It is largely cultivated in more than 80 countries worldwide, including China, India, the USA, and Pakistan (Zahra et al., 2019). However, it is commonly known to be susceptible to waterlogging stress (Bange et al., 2004). Waterlogging stress damages cotton plant growth and development, as well as nutrient uptake (Dodd et al., 2013), resulting in yield reduction (Cao et al., 2012; Najeeb et al., 2015). Existing research on cotton tolerance mechanisms has proven that cotton adapts to waterlogging using three primary mechanisms: escape, quiescence adaptation, and self-regulation and compensation (Zhang et al., 2020). The plant uses the escape strategy under short-term waterlogging stress by increasing adventitious root growth, producing aerenchyma, and accelerating stem elongation (Hussain et al., 2014). Self-regulation and compensation strategies include indeterminate growth habits, compensatory abilities, and the acceleration of plant growth and development after stress recovery (Sadras, 1995; Zhang et al., 2017). Finally, the quiescent adaptation strategy involves changes in the activity of protective enzyme systems, changes in hormone concentration and distribution, differential expression of genes (Zhang et al., 2020), and hydrogen peroxide H_2_O_2_ signaling (Zhao et al., 2018). In China, areas closer to the Yangtze River and the Yellow River along the middle and lower plains have high potential for supporting plantations of fast-growing plants in the future. However, these places are highly flooded (Gong et al., 2007). Moreover, in the context of China’s global climate change records, flooding is a frequently occurring abiotic stress, in addition to drought, which poses yearly threats to agricultural productivity (Qian et al., 2020). Furthermore, an extreme number of reports on cotton tolerance mechanisms have centered on escape strategies, self-regulatory strategies, and compensational waterlogging stress adaptability strategies (Yin et al., 2010). However, research on quiescence adaptability, which involves more molecular studies by coupling transcriptomics and metabolomics resistance mechanisms, is minimal. In addition, there is lesser information of cotton-tolerant responses to long-term waterlogging. Therefore, the objectives of this study were to observe the morphological changes in “CJ1831056” and “CJ1831072” cotton genotypes during long-term waterlogging stress and primarily the potential tolerance mechanisms of cotton at the transcriptome and metabolome profiles in response to waterlogging stress. To attain this goal, we first revealed induced waterlogging-responsive genes through the analysis of differentially expressed genes. Subsequently, we classified differentially expressed metabolites during long-term waterlogging using metabolomics analysis. Our results can provide a system-level context for advanced studies on the potential mechanism of waterlogging resistance in cotton and further contribute to the breeding of waterlogging-tolerant lines.

## Materials and methods

### Plant material

Two cotton (*Gossypium hirsutum* L.) lines “CJ1831056 and CJ1831072” were chosen for this study. Seed materials were sourced from the Institute of Cotton Research, Chinese Academy of Agricultural Sciences (ICRCAAS). Cotton seeds were planted in a controlled environment (greenhouse) under natural lighting and temperature (22.2°C/day and 18.2°C/night) and relative humidity ranging from 70% to 85%.

### Waterlogging stress treatment and sampling

After seed emergence, seedlings were treated with a modified Hoagland nutrient solution containing 5 mM nitrate ( ${\text{NO}}_{3}^{-}$ ), as described previously (Sharma et al., 2019). After 30 days of culturing seedlings, plants were screened at a 0-day treatment as a control and at 5-, 10, 15-, and 20-day treatments to determine the ideal time points for waterlogging for subsequent experiments, where pots were kept in 5-cm-deep tap water (waterlogged, WL). After waterlogging stress time-point screening, vigorous physio-morphological changes in cotton leaves, stems, and roots were observed at both 10 and 20 days of stress. Hence, control (CK) experiments at 0 days and treated groups at 10 and 20 days of waterlogging time points were used. The primary roots, lateral roots, and adventitious roots were collected from each plant, frozen separately in liquid nitrogen, and stored at −80°C.

### RNA-seq library preparation

A total of l µg of RNA per sample was used as the input material for the RNA sample preparations. Sequencing libraries were generated using the NEBNext® Ultra™ RNA Library Prep Kit for Illumina (NEB, USA) following the manufacturer’s recommendations. Index codes were added to attribute sequences to each sample. mRNA was purified from total RNA using poly-T oligo-attached magnetic beads. Fragmentation was carried out using divalent cations at elevated temperatures in NEBNext First Strand Synthesis Reaction Buffer (5×). First-strand cDNA was synthesized using random hexamer primers and M-MuLV Reverse Transcriptase. To synthesize the second cDNA strand, the first cDNA strand was combined with DNA Polymerase I and RNaseH. The remaining overhangs were converted into blunt ends via exonuclease and polymerase activities. After adenylation of the 3′ ends of the DNA fragments, the NEBNext Adaptor with a hairpin loop structure was ligated to prepare for hybridization. To select cDNA fragments that were preferentially 250–300 bp in length, the library fragments were purified with the AMPure XP system (Beckman Coulter, Beverly, USA). Then, 3 μl of USER Enzyme (NEB, USA) was used with size-selected, adaptor-ligated cDNA at 37°C for 15 min, followed by 5 min at 95°C before PCR. PCR was then performed with Phusion High-Fidelity DNA polymerase, universal PCR primers, and Index(X) Primer. Finally, PCR products were purified (AMPure XP system), and library quality was assessed using the Agilent Bioanalyzer 2100 system. The clustering of the index-coded samples was performed on a cBot cluster generation system using the TruSeq PE Cluster Kit v3-cBot-HS (Illumina), according to the manufacturer’s instructions. After cluster generation, the library preparations were sequenced on an Illumina NovaSeq platform, and 150-bp paired-end reads were generated.

### RNA-seq analysis

Reference genome and gene model annotation files were downloaded directly from the Cotton Genome website. The index of the reference genome was built using Hisat2 v2.0.5, and paired-end clean reads were aligned to the reference genome using Hisat2 v2.0.5. Feature Counts v1.5.0-p3 was used to count the read numbers mapped to each gene. The fragments per kilobase of exon per million mapped (FPKM) of each gene was calculated based on the length of the gene and the read count mapped to this gene. Based on the raw count data, differential expression analysis between samples was performed using the DESeq2 R package (1.16.1). The resulting P-values were adjusted using Benjamini and Hochberg’s approach to controlling the false discovery rate. Genes with an adjusted P-value<0.05 found by DESeq2 were assigned as differentially expressed.

### GO and KEGG enrichment analyses

Gene Ontology (GO) enrichment analysis of differentially expressed genes was implemented using the cluster Profiler R package, in which gene length bias was corrected. GO terms with a corrected P value of less than 0.05 were considered significantly enriched by differentially expressed genes. KEGG is a database resource for understanding high-level functions and utilities of the biological system, such as the cell, organism, and ecosystem, from molecular-level information, especially large-scale molecular datasets generated by genome sequencing and other high-throughput experimental technologies (http://www.genome.jp/kegg/). We used the cluster Profiler R package to test the statistical enrichment of differentially expressed genes (DEGs) in the KEGG pathways.

### Metabolite extraction, LC-MS/MS analysis, and metabolite quantification

For metabolite extraction, 20 mg of the sample was weighted onto an EP tube after grinding with freeze-drying, and 1000 μl of extract solution (methanol: water = 3: 1, with isotopically labeled internal standard mixture) was added. Then, the samples were homogenized at 35 Hz for 4 min and sonicated for 5 min in an ice water bath. The homogenization and sonication cycles were repeated three times. The samples were then incubated at −40°C for 1 h before being centrifuged at 12,000 rpm (RCF = 13,800*g*, R = 8.6 cm) for 15 min at 4°C. The resulting supernatant was transferred to a fresh glass vial for analysis. The quality control (QC) sample was prepared by mixing an equal aliquot of supernatants from all samples. LC-MS/MS analyses were performed using an UHPLC system (Vanquish, Thermo Fisher Scientific) with a UPLC HSS T3 column (2.1 mm × 100 mm, 1.8 μm) coupled to a Q Exactive HF-X mass spectrometer (Orbitrap MS, Thermo). To visualize group separation and find significantly changed metabolites, supervised orthogonal projections to latent structures-discriminate analysis (OPLS-DA) were applied. Then, a sevenfold cross validation was performed to calculate the values of R2 and Q2. R2 indicates how well the variation of a variable is explained, and Q2 indicates how well a variable can be predicted. To check the robustness and predictive ability of the OPLS-DA model, 200 permutations were conducted. Afterward, the R2 and Q2 intercept values were obtained. Here, the intercept value of Q2 represents the robustness of the model, the risk of overfitting, and the reliability of the model, which will be better the smaller the intercept value. Furthermore, in the OPLS-DA, the value of variable importance in projection (VIP) of the first principal component was obtained. It summarizes the contribution of each variable to the model. The metabolites with VIP >1 and P< 0.05 (Student t test) were considered significantly changed metabolites. In addition, commercial databases, namely, KEGG (http://www.genome.jp/kegg/) and MetaboAnalyst (http://www.metaboanalyst.ca/), were used for pathway enrichment analysis.

### Validation of RNA-seq by qRT-PCR

Quantitative real-time PCR (qRT-PCR) was conducted using six genes, namely, *XTH9*, *PER1*, *RAP2-3*, metallothionein (*MT1*), *RbohD*, and *ADH*. Respective qRT-PCR primers are listed in **Supplementary Table S6**. Total RNA was extracted from upland cotton fiber using TRIzol Reagent. cDNA synthesis was performed using an RT reagent kit (Tiangen, China). qRT-PCR was analyzed in a 20-μl reaction system (including 10 μl SYBR Premix Ex Taq™ II (2×), 2 μl cDNA, and 0.8 μl upstream and downstream primers) and a simple procedure (50°C for 2 min; 40 cycles at 95°C for 30 s, 95°C for 5 s, and 60°C for 20 s; and a final extension at 72°C for 10 min). Three biological repeats were included for each condition, and the GhUBQ gene was used as a control. The relative expression levels were calculated using the 2^-ΔΔCt^ method.

## Results

### Morphological variations of the two cotton genotypes to waterlogging stress

**Figure 1:**
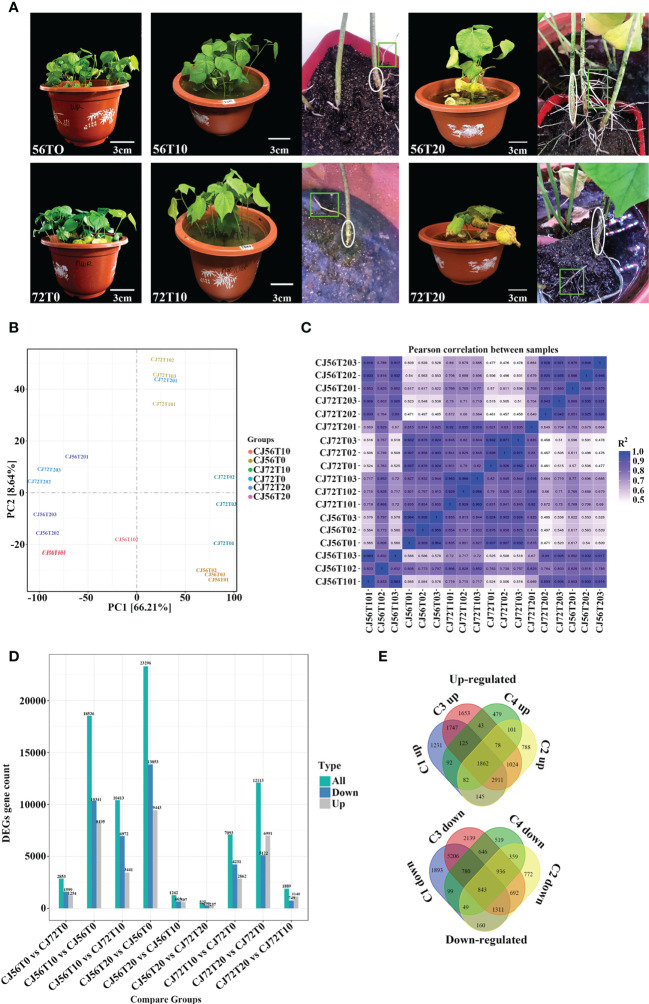
Observable morphological changes and gene expression analysis of “CJ1831072” and “CJ1831056” genotypes under waterlogging stress. **(A)** Cotton seedling morphological changes after 30 days of culture in 5 cm of water depth above the soil surface for 10 and 20 days, with 0 days as the control. Circles indicate hypertrophic lenticels, whereas squares/rectangles show adventitious roots. Scale bar: 3 cm. **(B)** Principal component analysis (PCA) between samples. **(C)** Correlation between samples obtained for cotton genotypes under the waterlogging treatment. The color of the box represents the degree of correlation; deep blue represents the highest degree of correlation, and light blue represents the lowest degree of correlation. **(D)** Total differentially expressed genes. **(E)** Venn diagram of both upregulated and downregulated genes. C1up/down, C2up/down, C3up/down, and C4up/down denote CJ656T10vsCJ56T0, CJ72T20vsCJ72T0, CJ56T20vsCJ56T0, and CJ72T10vsCJ72T0, respectively.

Morphological changes between CJ1831056 and CJ1831072 genotypes following 0 days of no stress and then 10 and 20 days of waterlogging stress were assessed. At 10 days of waterlogging compared with the control, the two cotton genotypes displayed green leaves and no signs of damage (**Figures 1A, B**). Formation of hypertrophic lenticel and few adventitious roots on cotton stems and roots were observed at 10 days of stress in both genotypes (**Figures 1A, B**). Although cotton leaves of both genotypes turned yellow and chlorotic at 20 days of stress, formation of hypertrophic lenticel and adventitious roots was vigorous in CJ1831056 and CJ1831072 (**Figures 1A, C**).

### Transcriptome analysis

Here, a total of 18 qualified libraries were established. Clean reads were obtained by removing low-quality reads. Approximately 800 million clean reads were obtained with a total of 120.07 Gb of sequence data. Clean readings at Q20 and Q30 were over 98% and 93%, respectively (**Supplementary Table 1**). Principal component analysis (PCA) revealed that replicated biological samples significantly clustered together. This shows that biological replicates, consistent samples, and sequencing results were reliable (**Figure 1B**). Pearson’s correlation coefficient (PC) analysis was performed to check the correlation between all samples (**Figure 1C**). Results show a higher correlation at 10 and 20 days of replicated samples compared with 0 days. Furthermore, transcriptomics differentially expressed genes revealed 101,599 genes. To effectively analyze and interpret the DEGs, a P-value< 0.05 and |log2 fold change| ≥2 were used to effectively analyze and interpret DEGs as visualized among comparisons (9) (**Figure 1D**). In general, CJ56T10 vs. CJ56T0 and CJ56T20 vs. CJ56T0 had 41,832 DEGs higher than 8,306 DEGs in CJ72T10 vs. CJ72T0, and CJ72T20 vs. CJ72T0 during waterlogging stress (**Figure 1D**). This shows that the CJ1831056 genotype has a stronger response to waterlogging than the CJ1831072 genotype at the transcription level. Moreover, 33,547 DEGs were assigned for gene regulation analysis using a Venn diagram. A total of 1,862 upregulated and 843 downregulated DEGs were shared among all four comparisons (**Figure 1E**). This revealed that upregulated genes were more responsive to waterlogging than downregulated genes. Hence, CJ56T10 vs. CJ56T0, CJ56T20 vs. CJ56T0, CJ72T10 vs. CJ72T0, and CJ72T20 vs. CJ72T0 comparisons were selected for further analysis. For GO analysis, the total annotated number of genes was 43,894, into three ontologies (**Supplementary Table 2****;** **Additional File 1**). In the biological process category, metabolic (17,784), cellular (15,675), and single-organism processes (10,731) were recorded to have a significantly expressed number of genes. The cellular component category was mainly cell (4,972), cell parts (4,972), and organelles (3,445). Molecular function categories included binding (25,575) and catalytic activity (17,034), as highly represented (**Supplementary Table 2**). For subsequent analysis, the assembled gene functions were evaluated through a search against the KOG database for functional prediction and classification (**Supplementary Table 2**).

### Pathway analysis of DEGs

**Figure 2:**
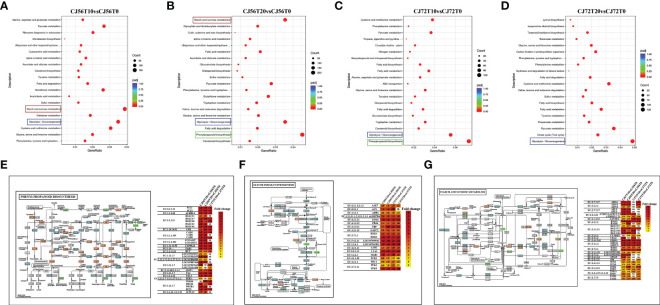
Pathway analysis. **(A–D)** KEGG enrichment analysis of top 20 most significant enrichment pathways in CJ1831056 and CJ1831072 at 10 and 20 days of stress. **(E)** Pattern diagram of the phenylpropanoid biosynthesis pathway with the relative expression level of DEGs. **(F)** Pattern diagram of the glycolysis and gluconeogenesis pathways with the relative expression level of DEGs. **(G)** Pattern diagram of the starch and sucrose metabolism pathways with the relative expression level of DEGs. Yellow color represents upregulated genes, red color represents downregulated genes, and blue represents both upregulated and downregulated genes. Respective relative expression levels of DEGs measured by log2 ratio (fold change between waterlogging treatment and control). Positive fold-change values (yellow) indicate upregulation, whereas negative fold-change values (red) indicate downregulation. ND indicates no detected expression.

Pathway analysis of DEGs was conducted by using the Kyoto Encyclopedia of Genes and Genomes (KEGG) pathway database. Various pathways were induced in cotton roots by waterlogging stress (**Supplementary Table 3**). The top enriched KEGG pathways among upregulated and downregulated DEGs in CJ1831056 and CJ1831072 are shown in **Supplementary Table 3**. A higher differentially expressed gene count was recorded in phenylpropanoid biosynthesis, glycolysis, gluconeogenesis, and starch and sucrose metabolism pathways (**Figures 2A–D** and **Supplementary Table 3**). The phenylpropanoid biosynthesis pathway, popularly known to regulate under hypoxia and waterlogging conditions (Zhang et al., 2017; Yu et al., 2020), revealed a higher expression of peroxidases (EC: 1.11.1.7) and beta glucosidases (EC: 3.2.1.21) (**Figure 2E**). This is a clear indication of ROS scavenging and activation of the plant defense system, hormone production, and cell wall lignification (Kongdin et al., 2021). In the glycolysis pathway, phosphofructokinase (EC: 2.7.1.11), alcohol dehydrogenase (EC: 1.1.1.1), and pyruvate decarboxylase-2 (EC: 4.1.1.1) genes were significantly expressed in CJ1831072 than in CJ1831056 (**Figure 2F**). Starch and sucrose metabolism pathways are known to be induced by waterlogging stress (Loreti et al., 2018). Similarly, starch synthase (EC: 2.4.1.21) SS4, sucrose synthase (EC: 2.4.1.13), chloroplast beta-amylase (EC: 3.2.1.2), and hexokinase (EC: 2.7.1.1) genes significantly responded to waterlogging in both cotton genotypes (**Figure 2G**).

### Effect of differentially expressed ROS gene and related antioxidant protection

**Figure 3:**
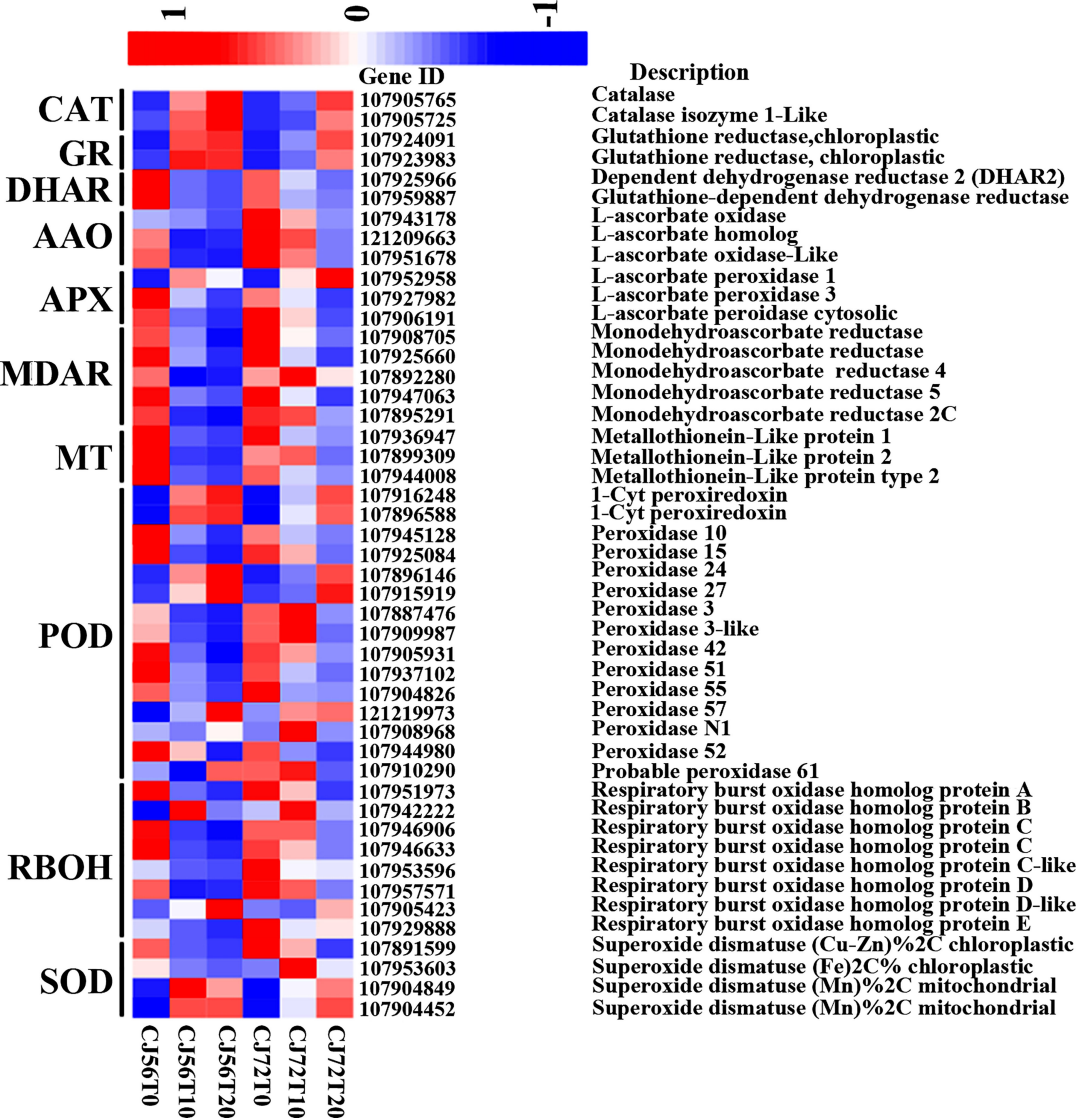
Heatmap analysis of DEGs involved in ROS and antioxidant signaling. The bar represents the scale of the expression levels of each gene (log2 FPKM) in each sample, as indicated by red/blue rectangles. Red rectangles represent the high expression of genes, and blue rectangles represent the low expression.

Identification of ROS and antioxidant and redox-sensing mechanisms plays a key role in waterlogging tolerance mechanisms. The ROS-generating *RBOH-D* (107905423) gene showed higher upregulation at 20 days of waterlogging in CJ1831056 than in CJ1831072 (**Figure 3**). Moreover, two *CAT* genes (107905765 and 107905725) were more upregulated in CJ1831056 than in CJ1831072 at 20 days of waterlogging, compared to the control (**Figure 3**). Peroxidase DEGs played a role in antioxidant signaling under waterlogging stress in cotton. For instance, 1-*Cys peroxiredoxin* (107916248) and *1-Cys peroxiredoxin-like* (107896588) were upregulated more after 10 and 20 days of waterlogging in both genotypes compared with the control. Four SOD genes were also induced in ROS scavenging in defense against waterlogging stress. Among DEGs involved in SOD, superoxide dismutase (FE) % chloroplastic 2C was more upregulated in CJ1831072 than in CJ1831056 at 10 days of waterlogging, whereas superoxide dismutase (MN) % mitochondrial was more upregulated in CJ1831056 than in CJ1831072. *GR* genes (107924091 and 107923983), *DHAR* genes (107925966 and 107959887), L-ascorbate oxidase (*AAO*) genes (107943178, 107951678, and 121209663), and L-ascorbate peroxidase (*APX*) genes were also induced by waterlogging in cotton roots (**Figure 3**).

### Analysis of DEGs for TFs induced by waterlogging

**Figure 4:**
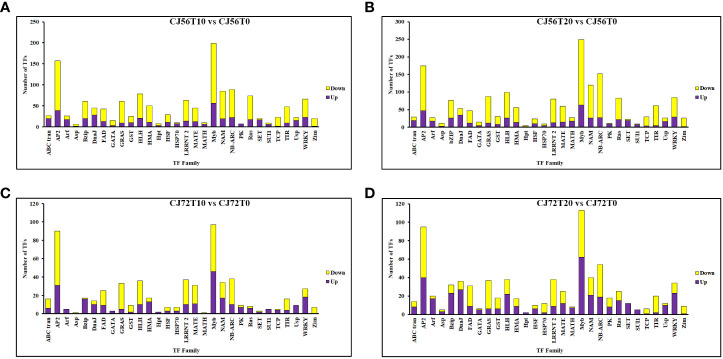
Differentially expressed transcription factor gene count in cotton roots during waterlogging. **(A)** CJ56T10 vs. CJ56T0 combination. **(B)** CJ56T20 vs. CJ56T0 combination. **(C)** CJ72T10 vs. CJ72T0 combination; **(D)** CJ72T20 vs. CJ72T0 combination.Yellow denotes downregulation, and purple denotes upregulation.

To further understand the potential tolerant mechanism of cotton toward genes waterlogging stress, highly responsive TFs were identified and analyzed. Interestingly, a considerable number of differentially expressed TFs were recorded. For instance, TFs such as *MYB DNA-binding*, *AP2*, *NB-ARC*, *NAM*, *bHLH*, *WRKY*, *LRRNT2*, *GRAS*, and *bZIP* were induced at 0, 10, and 20 days (**Figure 4**). However, among the two cotton lines, CJ1831056 genotypes showed higher recorded TFs than CJ1831072 genotypes. Parallel tendencies were also found, as DEGs downregulated were significantly higher than upregulated DEGs (**Figure 4**). Furthermore, among the TF families, differentially expressed *AP2* (2), *bZIP* (3), *bHLH* (1), *MYB* (1), *NAM* (1), and *WRKY* (2) were significantly upregulated in both genotypes. On the other hand, two *AP2*s, one *bZIP*, two *MYB*s, one *NAM*, and one *WRKY* gene were significantly downregulated (**Table 1**). However, similar genes of the same TF family showed a differential expression between the two genotypes.

**Table 1:** Comparisons of the expression levels of DEG transcription factors induced in “CJ1831056” and “CJ1831072” varieties. ‘ND’ represents not detected.

	Gene ID	56T10 vs. 56T0	56T20 vs. 56T0	Gene description	72T10 vs. 72T0	72T20 vs. 72T0
TF category		Log2FC	Log2FC		Log2FC	Log2FC
AP2	LOC107889672	5.7	6.5	Ethylene-responsive transcription factor ABR1-like	3.3	5.4
AP2	LOC107917284	5.0	5.1	Ethylene-responsive transcription factor ERF071	4.9	6.1
AP2	LOC121220769	−3.9	−6.8	AP2-like ethylene-responsive transcription factor	−8.0	−7.8
AP2	LOC107929210	−5.8	−2.0	Ethylene-responsive transcription factor ERF027-like	ND	−2.2
bZIP_1	LOC107913052	4.0	3.2	bZIP transcription factor 11-like	3.0	3.4
bZIP_1	LOC107901213	3.2	2.5	bZIP transcription factor 2-like	2.4	2.6
bZIP_1	LOC107946949	2.4	2.3	bZIP transcription factor 59	1.5	4.1
bZIP_1	LOC107951428	−3.4	−5.0	Transcription factor TGA3-like	ND	ND
bHLH	LOC107889526	3.2	3.3	Transcription factor bHLH130	2.0	3.1
Myb	LOC107946442	5.3	4.7	Transcription factor HHO2 (early flowering Myb)	1.7	3.4
Myb	LOC107889761	−3.7	−5.4	Transcription factor DIVARICATA	ND	ND
Myb	LOC107922466	−4.1	−6.2	Myb-related protein 308	ND	ND
NAM	LOC121231838	−4.0	−2.8	NAC domain-containing protein 4-like	ND	ND
WRKY	LOC107923243	3.4	2.7	Probable WRKY transcription factor 27	2.6	2.7
WRKY	LOC107918521	2.0	2.2	Probable WRKY transcription factor 57	1.3	2.7
WRKY	LOC107920274	−1.1	−1.3	Probable WRKY transcription factor 2	ND	−1.1

### Metabolome analysis of cotton root under waterlogging stress

**Figure 5:**
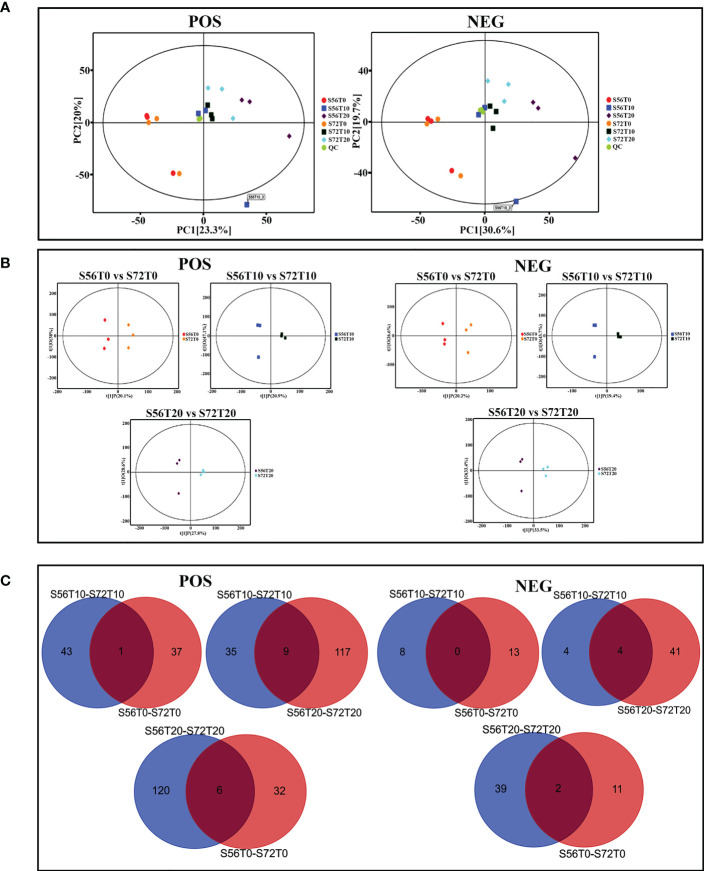
PCA score plot, OPLS-DA scatter plot, and Venn diagram analysis. **(A)** PC1 and PC2 coordinates for both POS and NEG coordinates. Each scatter point represents a sample, and the color and shape of the scatter point represent different groups. **(B)** OPLS-DA models of positive/negative ion modes for S56T0 vs. S72T0, S56T10 vs. S72T10, and S56T20 vs. S72T20. Ordinate t(1)P denotes the predicted principal component score for the first principal component, whereas ordinate t(1)O represents orthogonal principal component scores. All samples are within the 95% confidence interval (Hotelling’s T-square eclipse). **(C)** Venn diagram showing differentially upregulated and downregulated metabolites. POS denotes positive ion mode, and NEG denotes negative ion mode.

For the UHPLC system, OPLS-DA and PCA were used to reduce dimensionality from metabolomics data groups generated by LC-MS/MS analyses. The ionization source of the QE platform is electrospray ionization, which comprises both positive ion modes (POS) and negative ion modes (NEG). The explanation and predictability values were measured for the first two principal components (PCs) for POS (23.3% and 20%) and NEG (30.6% and 19.7%). In general, S56T10 and S72T10 have strong relationships with quality control (QC). However, the treated groups for 20 days were more significant in POS and NEG (**Figure 5A**). In addition, the OPLS-DA score chart differences between all groups of samples were significant (within the 95% confidence interval), confirming that the OPLS-DA results were valid (**Figure 5B**). The separation of control groups from treated groups and variation within treated groups indicate clear differences in metabolite accumulation between samples. Metabolite studies also show more differentially expressed metabolites (DEM) of 171 DEM at 20 days and 52 DEM at 10 days of stress compared to 51 DEM at 0 days of no stress in both POS and NEG metabolite detection coverage (**Figure 5C**). As shown in **Table 2**, top 20 significantly accumulated metabolites were selected by their respective fold change values and OPLS-DA methods (**Table 2**). Zero days of waterlogging stress revealed two significantly upregulated metabolites (dibutyl phthalate 1.6 FC and equol 1.1 FC) and one downregulated metabolite (15-KETE −1.5 FC) (**Table 2**). At 10 days of waterlogging, phenylpropanoids and polyketides (nobiletin −3.1 FC), organoheterocyclic (urocanic acid −2.3 FC), and benzenoids (sinapyl alcohol −1.8 FC) were significantly downregulated metabolites responsive to waterlogging. In contrast, an organic acid compound, galactaric acid 3.0 FC, was significantly upregulated at 10 days (**Table 2**). Moreover, 20 days of waterlogging revealed highly significantly upregulated metabolites including lipids and lipid-like molecules (gamma-linolenic acid 1.1 FC and testosterone 2.0 FC), organic acids, and derivatives (L-asparagine 1.5 FC, pyrrolidonecarboxylic acid 1.3 FC, and L-valine 1.1 FC) (**Table 2**). Conversely, significantly repressed metabolites responsive to 20 days of waterlogging were organoheterocyclic (E,E-trichostachine −3,6FC), nucleosides, nucleotides, analogue compound (adenosine monophosphate), organic acids and derivatives (N-acetyl-L-phenylalanine −1.9 FC and citric acid −1.7 FC), organic oxygen compounds (melibiose −1.2 FC), benzenoid (carmine red −2.0 FC), and phenylpropanoids and polyketides (procyanidin B2 −1.6 FC) (**Table 2**).

**Table 2:** Top 20 important POS/NEG metabolites with their kyoto encyclopedia of genes and genomes identifier number (KEGG ID/PubChem CID/Metabolite ID) and molecular formula (MF), identified through significant analysis of metabolites (SAM) and orthogonal partial least square discrepant analysis (OPLS-DA) in CJ1831056 and CJ1831072 cotton genotypes.

Stress time	Metabolite name	Compound ID	Compound class	Molecular formula	VIP	FC	Metabolite type
	Dibutyl phthalate	C14214	Benzenoids	C_9_H_7_NO	2.21	1.6	POS
Zero stress	Equol	C14131	Phenylpropanoids and polyketides	C_15_H_14_O_3_	2.07	1.1	POS
	15-KETE	C00457	Lipids and lipid-like molecules	C_20_H_30_O_3_	1.93	−1.5	NEG
10 days	Nobiletin	C10112	Phenylpropanoids and polyketides	C_21_H_22_O_8_	1.66	−3.1	POS
	Urocanic acid	C00785	Organoheterocyclic compounds	C_6_H_6_N_2_O_2_	2.00	−2.3	POS
	Sinapyl alcohol	C02325	Benzenoids	C_11_H_14_O_4_	1.36	−1.8	POS
	Galactaric acid	C00879	Organic oxygen compounds	C_6_H_10_O_8_	2.06	3.0	NEG
20 days	(E,E)-Trichostachine	C10174	Organoheterocyclic compounds	C_16_H_17_NO_3_	1.54	−3.6	POS
	L-Asparagine	C00152	Organic acids and derivatives	C_4_H_8_N_2_O_3_	1.71	1.5	POS
	L-Valine	C00183	Organic acids and derivatives	C_5_H_11_NO_2_	1.89	1.1	POS
	Adenosine monophosphate	C00020	Nucleosides, nucleotides, and analogues	C_10_H_12_N_5_O_6_P	1.52	−2.7	NEG
	N-Acetyl-L-phenylalanine	C03519	Organic acids and derivatives	C_11_H_13_NO_3_	1.01	−1.9	NEG
	Citric acid	C00158	Organic acids and derivatives	C_6_H_8_O_7_	1.15	−1.7	NEG
	Gamma-linolenic acid	C06426	Lipids and lipid-like molecules	C_18_H_30_O_2_	1.56	1.1	NEG
	Carmine red	C11254	Benzenoids	C_22_H_20_O_13_	1.32	−2.0	NEG
	Procyanidin B2	C17639	Phenylpropanoids and polyketides	C_30_H_26_O_12_	1.30	−1.8	POS
	Melibiose	C05402	Organic oxygen compounds	C_12_H_22_O_11_	1.70	−1.2	POS
	Testosterone	C00535	Lipids and lipid-like molecules	C_19_H_28_O_2_	2.21	2.0	POS
	Pyrrolidonecarboxylic acid	C02237	Organic acids and derivatives	C_5_H_7_NO_3_	1.62	1.3	NEG

### Correlation analysis between DEGs and metabolite in phenylpropanoid biosynthesis during waterlogging

**Figure 6:**
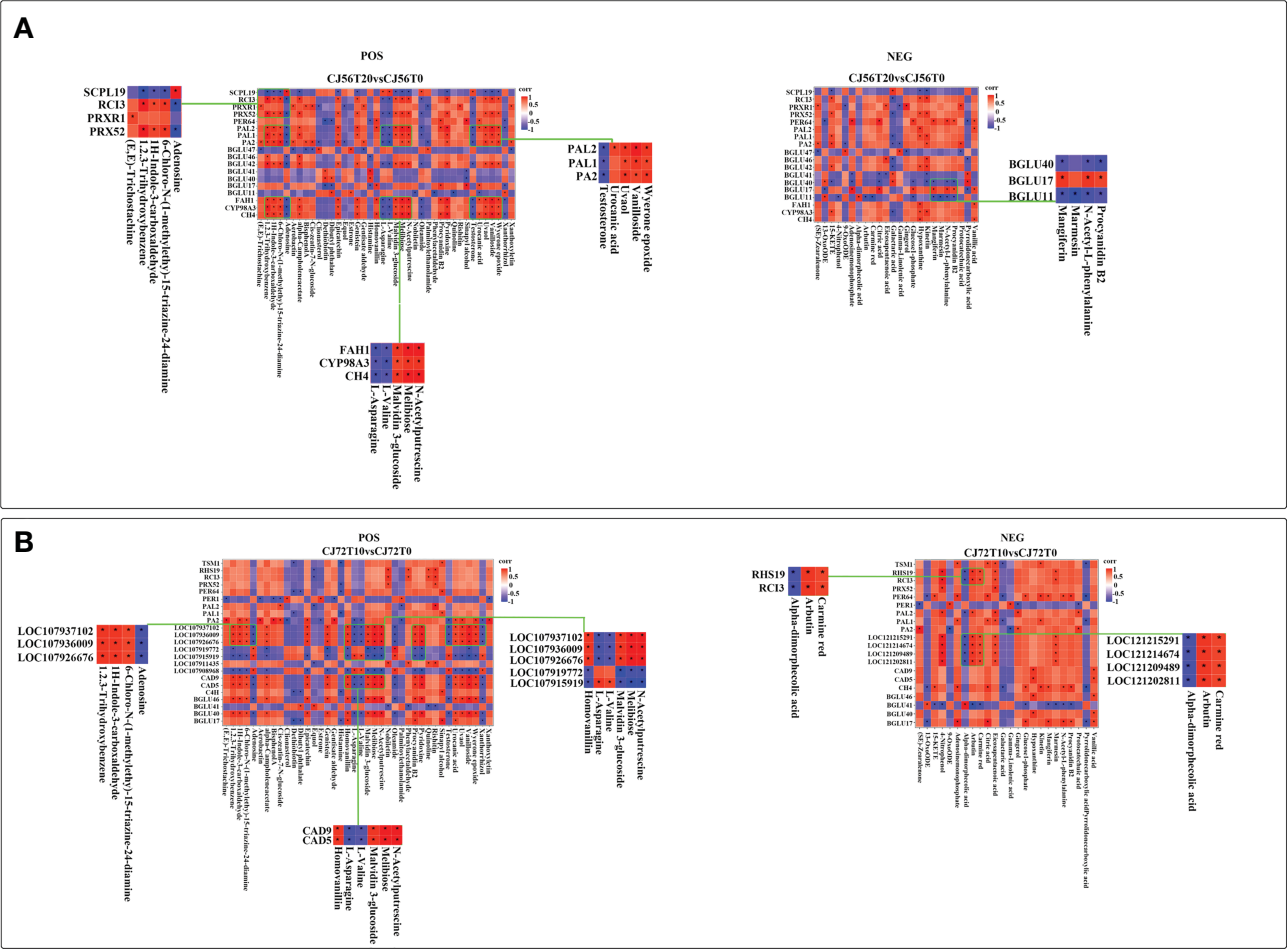
Spearman correlation analysis of DEGs in the phenylpropanoid biosynthesis pathway–accumulated POS and NEG metabolite modes. **(A)** Correlation between DEGs in CJ56T20 vs. CJ56T0 and metabolites in POS and NEG modes. **(B)** Correlation between DEGs in CJ72T10 vs. CJ72T0 and metabolites in POS and NEG modes. Each square of the heat map indicates a correlation coefficient score resulting from a Spearman correlation coefficient of 0.89. Red indicates a positive correlation, whereas blue indicates a negative correlation. An asterisk * indicates a significant correlation.

To investigate the relationship of DEGs and differentially accumulated metabolites in cotton under long-term waterlogging, a correlation analysis was performed between DEGs in CJ56T20 vs. CJ56T0 and CJ72T10 vs. CJ72T0 and between POS and NEG highly expressed metabolites with their respective KEEG ID. The correlation of each DEG–metabolite pair was assessed at a correlation coefficient of 0.89. The details of the representative phenylpropanoid DEG–metabolite correlation analysis are listed in Additional file 2. In addition, the correlations of representative DEG–metabolites are visualized in **Figure 6**. In CJ56T20 vs. CJ56T0, POS metabolites correlated with DEGs; adenosine, L-asparagine, L-valine, and testosterone metabolites had a considerably negative correlation with DEGs, such as *PA2*, *PAL1*, *PAL2*, *BGLU42*, *C4H*, *CYP98A3*, and *FAH1*. In contrast, melibiose, positively correlated with *PRX52* whereas sinapyl alcohol correlated positively with *PER64* (**Figure 6A**). In the NEG CJ56T20 vs. CJ56T0 comparison with metabolites, galactaric acid forms a significant negative correlation with DEGs, such as *CYP98A3*, *BGLU42*, *BGLU46*, *PAL1*, *PA2*, *PRX52*, and *RCI3* (**Figure 6A**). Conversely, hypoxanthine metabolites formed significant positive correlations with *CYP98A3*, *BGLU42*, *PAL1*, *PRX52*, and *RCI3* (**Figure 6A**). Furthermore, in CJ72T10 vs. CJ72T0, DEGs *BGLU40*, *BGLU46*, *107937102*, *107936009*, *107926676*, *CAD9*, and *CAD5* were significantly positively correlated with procyanidin B2 metabolite. However, these DEGs were negatively correlated with adenosine, L-valine, L-asparagine, and testosterone metabolites (**Figure 6B**). Conversely, *RHS19*, *RCI3*, *121215291*, *121214674*, *121209489*, and *121202811* DEGs significantly positively correlated with metabolites, carmine red (**Figure 6B**).

### DEGs validation by qRT-PCR

**Figure 7:**
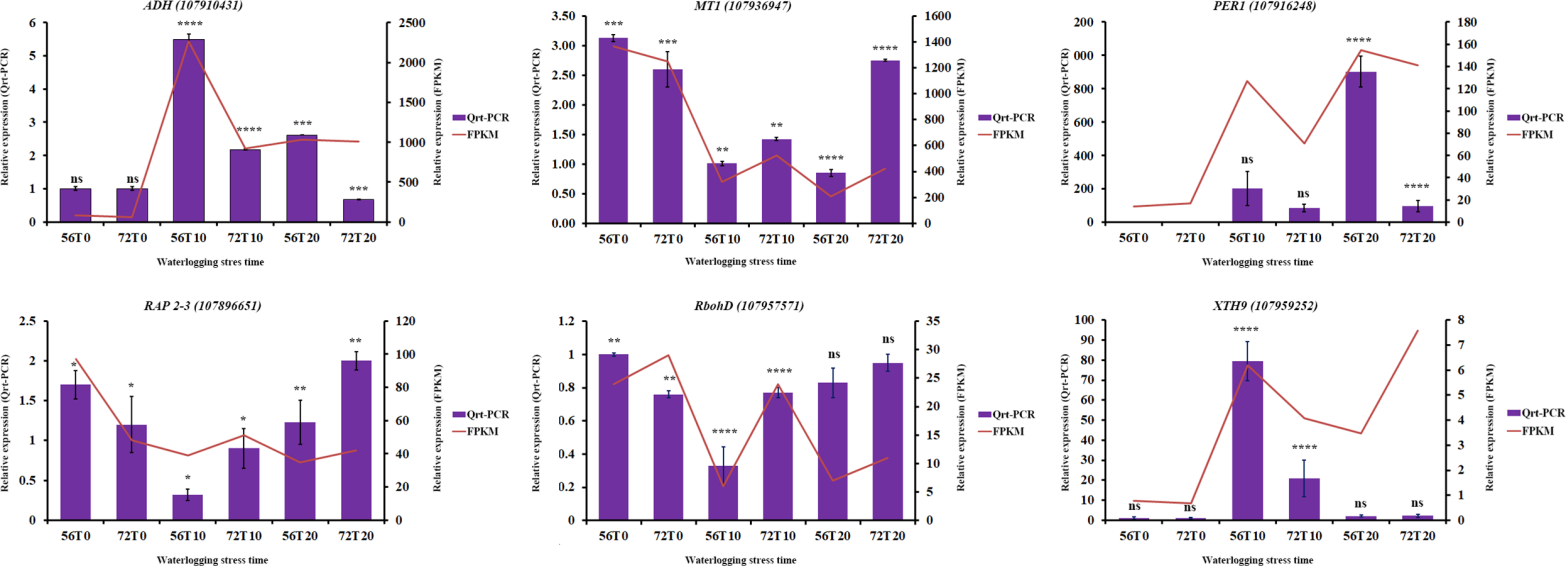
qRT-PCR verification of some key genes with RNA-seq expression. Bars represent qRT-PCR expression, and zigzags represent FPKM expression. Each bar represents the mean ± SD. Asterisk *, ***, and **** represent the significant differences (P< 0.1 to<0.05 two-way ANOVA with Tukey’s HSD post-hoc test), whereas ns indicates no significance, respectively. 56T0/72T0, 56T10/721T0, and 56T20/72T20 denote 0 days of no waterlogging, 10 days of waterlogging, and 20 days of waterlogging, respectively.

To verify the accuracy of the DEGs, qRT-PCR analysis was conducted to compare the consistency in the expression of some genes (Wang et al., 2022). Six differentially expressed genes, namely, *ADH-class-P-like* (107910431), *PER1* (107916248), *RbohD* (107957571), *RAP2-3* (107896651), *MT1* (107936947), and *XTH9* ((107959252), were randomly chosen (**Figure 7**). As shown in **Figure 7**, *ADH-class-P-like* and *XTH9* genes had significant expression levels during 10 days of waterlogging stress in CJ1831056 than in CJ1831072, whereas the relative expression levels in *RbohD*, *RAP 2-3*, and *MT1* were significantly higher in CJ1831072 than in CJ1831056 (**Figure 7**). In addition, the relative expression levels of *PER1* and *ADH-class-P-like* were significantly higher at 20 days of waterlogging in CJ1831056 than in CJ1831072, whereas in *RAP 2-3* and *MT1* genes at 20 days of waterlogging, significant expressions were observed in CJ1831072 than CJ1831056 (**Supplementary Table 6** and **Figure 7**). The expression patterns of five out six genes were consistent with RNA-seq data (**Figure 7**). These results highly confirm the credibility of the RNA-seq results.

## Discussion

### Morphological tolerant mechanisms in waterlogged cotton

Plants’ tolerance against flooding has always been extensively studied. However, the ability of plants to form adventitious roots and hypertrophic lenticel are major keys to tolerance against flooding (Else et al., 2009; Shimamura et al., 2010). Formation of adventitious roots at the hypocotyl internodes or at the stem base during waterlogging stress facilitates gas exchange and nutrition absorption (Qi et al., 2019). To a greater extent, these root tissues usually replace primary roots that die as a result of hypoxic stress, allowing normal growth and development to progress (Eysholdt-Derzsó and Sauter, 2019). In the present investigation, vigorous formation adventitious roots were found on waterlogged cotton root and stems. However, formation was numerous in the CJ1831056 genotype at 20 days of stress (**Figure 1A**). This illustrates that waterlogged cotton may use root and stem adventitious root formation as an escape tolerance strategy for subsequent respiration. Moreover, hormones play a vital role in adventitious root growth in plants’ tolerance against waterlogging. For example, in cucumber, both auxins and ethylene were found to induce adventitious root growth under waterlogged conditions (Qi et al., 2019). Similarly, our present study showed a higher expression of differentially expressed auxins and ethylene genes in CJ1831056 genotypes in comparison with CJ1831072 genotypes (**Figure S1**). We hypothesized that both hormones may have a significant role in cotton tolerance against waterlogging stress. A recent report showed that a putative cell-wall-loosening enzyme xyloglucan endotransglucosylase/hydrolase (XTH) may be involved in cell wall metabolism during flooding-induced aerenchyma and adventitious root growth. Although the *XTH* gene family is thought to play a major role in cell wall remodeling, it is also proven to play a significant role in plants’ tolerance against abiotic stresses such as flooding (Song et al., 2018). For instance in barley, upregulation of xyloglucan endotransglucosylase/hydrolase in roots improves waterlogging resistance (Luan et al., 2018). In cotton, Gh*XTH1* and Gh*XTH3* were upregulated in the roots of tolerant waterlogged genotypes (Zhang et al., 2020). Similarly in transgenic soybean, overexpression of the *AtXTH31* gene induced more adventitious roots in tolerance against flooding stress at the early seedling growth stage (Song et al., 2018). Clearly, our results showed the expression of four *XTH* DEGs (*XTH9*, *XTH22*, *XTH30*, and *XTH21*) (**Supplementary Table 4**). We hypothesized that XTH-mediated cell wall adjustment may have a role in cotton adaptation to waterlogging stress, along with a useful gene family to develop flooding tolerance lines through molecular transgenic breeding study. Hypertrophic lenticel formation is a sign of tolerance in flooding conditions. Previous reports show that it regulates oxygen transport to the root by acting as a conduit through the cambium layer for gaseous uptake (Le Provost et al., 2016). As shown in **Figure 1**, 20 days of stress showed vigorous lenticel organs in the CJ1831056 cotton genotype than CJ1831072. This shows that induction of hypertrophic lenticel organs may play a vital role in cotton tolerance against waterlogging stress.

### Mechanism of ROS and antioxidant tolerance in waterlogged cotton

The incidence of abiotic stress activates ROS overproduction. ROS accumulation is usually regulated by the respiratory burst homolog D (*RbohD*) which enhances ROS production and then acts as a sensory signal for other cells to increase their ROS production (Mansoor et al., 2022). In high quantities, ROS negatively affects plant growth but, when minimized, is capable of inducing defense and hypersensitive responses and protein signaling (Qi et al., 2019; Fardus et al., 2021). In the present study, *RhohD* (107957571) was induced by waterlogging in both genotypes. However, it was more repressed in CJ1831056 than in CJ1821072 (**Figure 3**), indicating that ROS production may have played a significant role in waterlogged cotton tolerance, and moreover, CJ1831056 may have a tendency to withstand waterlogging stress by repressing ROS accumulation. A clear signal of high production of harmful ROS substance is proportional to the high production of antioxidant scavenging enzymes. In the present study, antioxidant scavenging enzymes such as PODs, DHARs, SODs, MDHAR, AAOs, CATs, MTs and APXs significantly expressed to reduce the harmful effects of ROS (**Figure 3**). According to existing reports, the CAT enzyme catalyzes the conversion of the ROS compound (H_2_O_2_) into water and oxygen (Kaushal et al., 2018). In addition, SOD is popularly known to remove ROS in anaerobic organisms based on the type of metal cofactor, and POD also catalyzes the reduction of hydrogen peroxide and alkyl hydroperoxides against oxidative damage (Nevalainen, 2010; Cheng et al., 2015). Clearly, this present study showed significant expressions of CAT and POD DEGs at 10 and 20 days in CJ1831056 than in CJ1831072, respectively (**Figure 3**). In addition, *MnSOD* and *FeSOD* gene expressions were significant in CJ1831056 than in CJ1831072 at 10 days of waterlogging, but the *FeSOD* gene was poorly induced at 20 days in both genotypes (**Figure 3**). We hypothesized that this could contribute to waterlogging resistance in the CJ1831072 genotype at 10 days of waterlogging (**Figure 1A**). Metallothionein (MT) is a small cysteine-rich protein that plays a role in ROS scavenging in biotic and abiotic stresses (Patankar et al., 2019). Although the role of MTs in waterlogging resistance is minimal, in maize, downregulation of the *MT1* gene is thought to play a role in the regulation of aerenchyma formation in the root cortex (Rajhi et al., 2011). Similarly, in the present study, *MT1* was highly downregulated in both genotypes, with a higher expression in the CJ1831056 at the designated waterlogging time points (**Figure 3**). Our findings clearly suggest that MTs may not only play a role in ROS scavenging but also determines the fate of inducible aerenchyma formation in cotton genotypes under waterlogging stress. Sucrose synthase, catalyzing sucrose to UDP-glucose and fructose, plays a fundamental role in providing an adequate sugar supply during anoxic stress, Zeng and colleagues recently demonstrated that waterlogging for 5–10 days reduced sucrose synthase activities, most likely due to changes in gene expression in starch and sucrose metabolism (Zeng et al., 2021). Contrarily, the presence of sucrose in anoxic conditions significantly alleviated meristem death and was capable of restoring root tip viability (Springer et al., 1986). Here, the starch and sucrose pathways were significantly expressed, and related genes were significantly downregulated in both genotypes (**Figure 2G** and **Figure S2B**). We speculated that the downregulation of numerous sucrose synthase genes in response to waterlogging may have played a role in root chlorosis and leaf dropping in cotton genotypes (**Figure 1A**).

### Differentially expressed TF responses in waterlogged cotton roots

Stress-responsive TFs play critical roles in abiotic stress responses and waterlogging stress tolerance in plants including members of the *AP2/ERF*, *MYB*, *bHLH*, *NAC*, *WRKY*, and *bZIP* families (Yoon et al., 2020; Zhang et al., 2022). Similar expressions of these TFs were discovered in this study (**Figure 4**). N-end pathway TFs are popularly known as oxygen-sensing mechanisms thought to trigger responses that destabilize proteins in oxygen-deprived environments (Perata and Dongen, 2011). Key N-end rule pathway DEGs, such as *RAP2.12*, *RAP2.3*, *HRE2*, *PCO1*, and *PCO2*, were upregulated by waterlogging and downregulated by reoxygenation in both tolerant and resistant chrysanthemum cultivars (Zhao et al., 2018). However, in the present study, all *RAP2-3* and *RAP2-12* DEGs were significantly upregulated at 0 days of no stress but repressed at waterlogging for 10 and 20 days (**Figure S2**). Based on the present results, we speculated that VII ERFs played a significant role in tolerance, but expression could be relative to the plant species. PCO activity is known as a signaling response to changes in oxygen availability. According to recent reports, during submergence, PCO activity drops, resulting in increased stabilization of ERF-VIIs (Taylor-kearney and Flashman, 2021). However, in this study, PCO DEGs were highly expressed during waterlogging stress but suppressed at no stress in CJ1831056, compared with CJ1831072. We speculated that the higher expression of PCO genes could be a result of ERF-VII instability. Among other TFs, APETALA2 (AP2) domain *ABR1*-like genes and ethylene-responsive transcription factor *ERF071* were significantly induced. *ABR1* acts as a repressor of ABA (Chen et al., 2020). However, *ERF71* is believed to contribute to tolerance in anoxia stress condition by increasing anaerobic gene expression and adventitious root growth under hypoxia (Licausi et al., 2010; Eysholdt-Derzsó and Sauter, 2017). In this study, both *ABR1*-like and *ERF71* genes were significantly upregulated (**Table 1**). We hypothesized that both genes may play a significant role in cotton tolerance to waterlogging in ABA repression and adventitious root growth. WRKY TFs play important roles, mainly in the innate immune system of plants. For instance, recent reports have shown that the expression of *GhWRKY27* was induced by leaf senescence in early-aging cotton (Gu et al., 2019). A similar result in this study showed that the WRKY27 gene was more expressed in the CJ1831056 than CJ1831072 genotype at 10 days of waterlogging, but at 20 days of waterlogging, expression in the CJ1831056 genotype decreased as expression in the CJ1831072 genotype increased (**Table 1**). This shows that the WRKY27 senescence gene was significantly enhanced in CJ1831072 compared with CJ1831056, as waterlogging time increased.

### Responsive metabolites in waterlogged cotton root

Metabolomics studies in cotton genotypes, CJ1831056 and CJ1831072, significantly induced phenylpropanoid biosynthesis, purine metabolism, and galactose metabolism pathways (**Supplementary Table 5**). Zhang et al. (2021) explained that phenylpropanoid biosynthesis and purine metabolism are the major pathways that respond to abiotic conditions (Zhang et al., 2021). Similar to the current investigation, phenylpropanoid biosynthesis and purine metabolism pathways were highly influenced at the metabolite level of study in responses to waterlogging stress. Moreover, adenosine and sinapyl alcohol metabolites were highly accumulated in these pathways at 10 days of waterlogging (**Supplementary Table 3**). Sinapyl alcohol metabolite acts as an antioxidant against oxidative injury (Takahama et al., 1996). In the present study, sinapyl alcohol was upregulated in CJ1831072 but downregulated in CJ1831056 (**Figure S3B**). We speculated that the early accumulation of sinapyl alcohol may play a role in the phenotypic resistance observed at 10 days of stress (**Figure 1B**). Adenosine, produced from hypoxic and damaged tissues, reduces proinflammatory activities by facilitating anti-inflammatory activities (Ohta, 2016). Adenosine metabolite accumulation observed in this study was upregulated in CJ1831056 but downregulated in CJ1831072 (**Figure S3B**). First, we hypothesized that accumulation of adenosine was triggered by ROS in both genotypes at 10 days of waterlogging; however, it had high-tolerance responses in CJ1831056 than CJ1831072.

Furthermore, two metabolites, galactaric acid and nobiletin, were highly accumulated metabolites under 10 days of waterlogging, with fold change values of 3.0 and −3.1, respectively (**Figures S3B**, **S4B**). Galactaric acid has been shown to be involved in osmoprotection and stress signaling under drought (Marczak and Augustyniak, 2016). Therefore, its upregulation in CJ1831056 and downregulation in CJ1831072 may support the notion that the CJ1831056 genotype was protected from osmotic stress more than CJ1831072 (**Figure S4B**). Wang et al. (2018b) further reported that nobiletin phytochemicals protect *Saccharomyces cerevisiae* cells from the oxidative damage imposed by H_2_O_2_ (Wang et al., 2018b). We speculated that nobiletin metabolites also contributed to antioxidant protection at 10 days of waterlogging in cotton. However, its expression was downregulated in CJ1831056 and upregulated in CJ1831072 (**Figure S4B**). In response to several kinds of abiotic stressors, plants greatly increase their accumulation of free amino acids and sugar compounds. In this study, valine, leucine, and isoleucine biosynthesis and galactose metabolism pathways were the two main pathways that responded to 20 days of waterlogging in cotton (**Supplementary Table 5**). Amino acid accumulation is evident in enhancing stress elasticity in plants (Fardus et al., 2021). In this study, L-glutamic acid, L-asparagine, and L-valine metabolites were induced at 20 days of waterlogging (**Figures S3C** and **S4C**). Reports have confirmed that exogenous applications of glutamate and glutamic acid enhance drought and salt tolerance in radish and Lens culinaris Medik., respectively (Fardus et al., 2021). These findings suggest that L-glutamic acid may play a role by increasing stress tolerance in plants, indicating that L-glutamic acid metabolite accumulation might regulate “arginine and proline metabolism” and “glutathione” pathways in this study (**Supplementary Table 5**). Additionally, at 20 days of waterlogging, amino acid metabolites, such as malic acid and citric acid, were both upregulated in CJ1831072 and downregulated in CJ1831056. Other metabolites, such as vanillic acid, gamma-linolenic acid, procyanidin C1, 9-OxoODE, and procyanidin B2, also accumulated significantly at 20 days of waterlogging (**Figure S4C**).

## Conclusion

**Figure 8:**
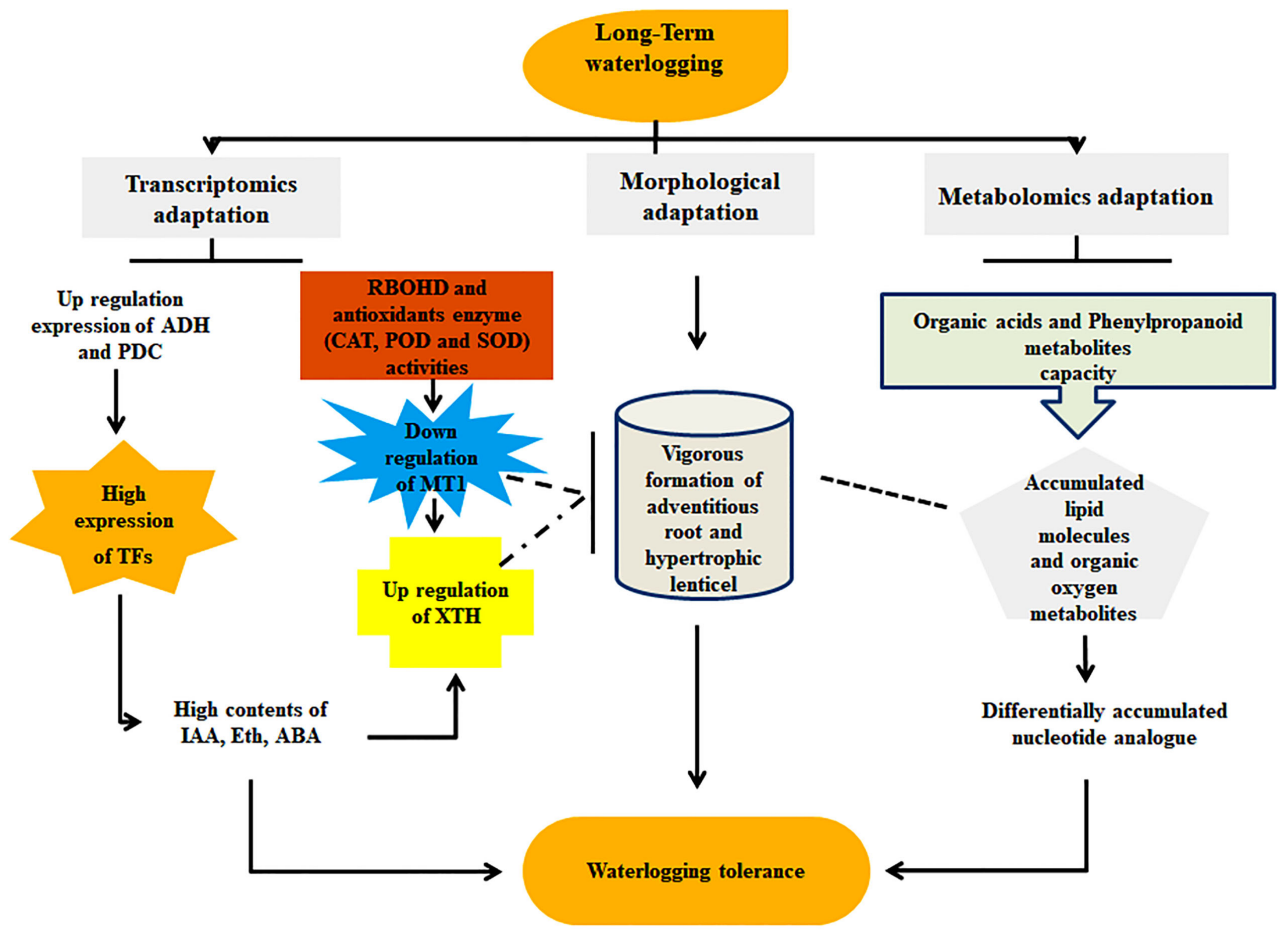
Molecular and morphological adaptation strategies of cotton in response to waterlogging stress. Molecular adaption denoted transcriptomic expression of genes (ADH: alcohol dehydrogenase, PDC: pyruvate decarboxylase, XTH: xyloglucanendo-transglucosylase/hydrolase, RBOHD: respiratory burst oxidase homolog protein D, CAT: catalase, SOD: superoxide dismutase, POD: peroxidase, MT1: metallothionein 1, TFs: transcription factors, IAA: indole-3-acetic acid, Eth: ethylene and ABA: abscisic acid) and metabolic expression of metabolites (organic acid, phenylpropanoid, organic oxygen, and nucleotide analogue). Morphological adaptation represents adventitious roots and hypertrophic lenticels formation.

The combination of metabolomic and transcriptomic analyses revealed the potential mechanism of long-term waterlogging resistance between the cotton genotypes CJ1831056 and CJ1831072. Transcriptomic analysis with RNA-seq revealed significant genes such as *PER1*, *PRX52*, *PER64*, *ADH*, *PDC*, *MT1*, *XTH*, and *SUS* to be the key genes highly contributing to waterlogging resistance and highly expressed in CJ1831056 than in CJ1831072. Moreover, innately resistant TFs, including *WRKY*, *AP2/ERF*, and *MYB*, were significantly induced in waterlogged CJ8131056 genotype roots, which hypothetically show signs of tolerance in response to waterlogging. Contrarily, metabolomics studies with metabolites revealed significant variations between the two genotypes after 10 to 20 days of waterlogging duration. Stress-tolerant metabolites, including sinapyl alcohol, adenosine, L-glutamic acid, galactaric acid, nobiletin, and L-asparagine, were significantly expressed in CJ1831056 than in CJ1831072 in response to long-term waterlogging. Based on these findings, a schematic model of cotton adaptable mechanisms to long-term waterlogging durations (**Figure 8**) is presented. These analyses provide comprehensive knowledge on the molecular understanding of cotton’s response to long-term waterlogging, providing effective guidance for future breeding techniques in cotton production.

## Data availability statement

The original contributions presented in the study are publicly available. This data can be found here: NCBI, PRJNA911339.

## Author contributions

Conceived and designed the experiments: J-SG and AGO. Experiments: AGO and Y-PL. Data analysis: AGO, J-SG, Y-PL, ML, YW, C-LL, NG, and D-HL. Main authors: AGO, Y-PL, and J-SG. Manuscript revision: J-SG and AGO. All authors contributed to the article and approved the submitted version.

## Supplementary material

Supplementary Figure 1 Number of hormone DEGs in cotton roots during waterlogging stress. (A) DEGs in CJ56T10vsCJ56T0. (B) DEGs in CJ56T20vsCJ56T0. (C) DEGs in CJ72T10vsCJ72T0. (D) DEGs in CJ72T20vsCJ72T0.

Supplementary Figure 2 Heat map clustering analysis of DEGs. **(A)** Clustering analysis of DEGs involved in the N-end rule pathway. **(B)** Clustering analysis of DEGs involved in carbohydrate metabolism. The bar represents the scale of the expression levels of each gene (log2 FPKM) in each sample, as indicated by red/blue rectangles. Red rectangles represent the high expression of genes, and blue rectangles represent the low expression.

Supplementary Figure 3 Analysis of accumulated metabolites at 10 days of waterlogging in cotton waterlogged roots. **(A)** Metabolites absolute value of the fold change. **(B)** The bubble plot represents a metabolic pathway. **(C)** Hierarchical cluster analysis. The abscissa represents the different experimental groups, the ordinate represents the comparative metabolites of the group, and the color blocks at different positions represent the relative expression amount of the metabolites at the corresponding positions. Red indicates high expression of the substance, and blue indicates low expression.

Supplementary Figure 4 Analysis of accumulated metabolites at 20 days of waterlogging in cotton waterlogged roots. **(A)** Metabolites’ absolute value of the fold change (waterlogging/Control) at 20 days in cotton roots. **(B)** The bubble plot represents a metabolic pathway. **(C)** Hierarchical cluster analysis. The abscissa represents the different experimental groups, the ordinate represents the comparative metabolites of the group, and the color blocks at different positions represent the relative expression amount of the metabolites at the corresponding positions. Red indicates high expression of the substance, and blue indicates low expression.

## Appendix A. Supporting information

Additional supporting information may be found online in the supporting information section at the end of the article.

## References

[B1] Bange M. P. Milroy S. P. Thongbai P. (2004). Growth and yield of cotton in response to waterlogging. F. Crop Res. 88, 129–142. doi: 10.1016/j.fcr.2003.12.002

[B2] Blokhina O. B. Chirkova T. V. Fagerstedt K. V. (2001). Anoxic stress leads to hydrogen peroxide formation in plant cells. J. Exp. Bot. 52, 1179–1190. doi: 10.1093/jxb/52.359.1179 11432936

[B3] Broughton S. Zhou G. Teakle N. L. (2015). Waterlogging tolerance is associated with root porosity in barley (Hordeum vulgare l). Mol. Breed. 35, 1–15. doi: 10.1007/s11032-015-0243-3

[B4] Cao G. Wang X. Liu Y. Luo W. (2012). Effect of water logging stress on cotton leaf area index and yield. Proc. Eng. 28, 202–209. doi: 10.1016/j.proeng.2012.01.706

[B5] Chávez-Arias C. C. Gómez-Caro S. Restrepo-Díaz H. (2019). Physiological, biochemical and chlorophyll fluorescence parameters of physalis peruviana l. seedlings exposed to different short-term waterlogging periods and fusarium wilt infection. Agronomy 9, 1–20. doi: 10.3390/agronomy9050213

[B6] Chen L. Zhang D. Song C. Wang H. Tang X. Chang Y. (2020). Transcriptomic analysis and specific expression of transcription factor genes in the root and sporophyll of dryopteris fragrans (L.) schott. Int. J. Mol. Sci. 21, 1–20. doi: 10.3390/ijms21197296 PMC7583955 33023244

[B7] Cheng X. Yu M. Zhang N. Zhou Z. Xu Q. Mei F.. (2015). Reactive oxygen species regulate programmed cell death progress of endosperm in winter wheat ( triticum aestivum l .) under waterlogging. Springer 2, 1–17. doi: 10.1007/s00709-015-0811-8 25854793

[B8] Coutinho I. D. Henning L. M. M. Döpp S. A. Nepomuceno A. Moraes L. A. C. Marcolino-Gomes J.. (2018). Flooded soybean metabolomic analysis reveals important primary and secondary metabolites involved in the hypoxia stress response and tolerance. Environ. Exp. Bot. 153, 176–187. doi: 10.1016/j.envexpbot.2018.05.018

[B9] Dodd K. Guppy B C. N. Lockwood P. V. Rochester I. J. (2013). Impact of waterlogging on the nutrition of cotton (Gossypium hirsutum l.) produced in sodic soils. Crop Pasture Sci. 64, 816–824. doi: 10.1071/CP13093

[B10] Else M. A. Janowiak F. Atkinson C. J. Jackson M. B. (2009). Root signals and stomatal closure in relation to photosynthesis, chlorophyll a fluorescence and adventitious rooting of flooded tomato plants. Ann. Bot. 103, 313–323. doi: 10.1093/aob/mcn208 19001430 PMC2707317

[B11] Eysholdt-Derzsó E. Sauter M. (2017). Root bending is antagonistically affected by hypoxia and ERF-mediated transcription *via* auxin signaling. Plant Physiol. 175, 412–423. doi: 10.1104/pp.17.00555 28698356 PMC5580755

[B12] Eysholdt-Derzsó E. Sauter M. (2019). Hypoxia and the group VII ethylene response transcription factor HRE2 promote adventitious root elongation in arabidopsis. Plant Biol. 21, 103–108. doi: 10.1111/plb.12873 29996004 PMC6585952

[B13] Fardus J. Hossain S. Fujita M. (2021). Modulation of the antioxidant defense system by exogenous l -glutamic acid application enhances salt tolerance in lentil ( lens culinaris medik .). Biomolecules 11, 1–15. doi: 10.3390/biom11040587 PMC8073835 33923634

[B14] Gong J. R. Zhang X. S. Huang Y. M. Zhang C. L. (2007). The effects of flooding on several hybrid poplar clones in northern China. Agrofor. Syst. 69, 77–88. doi: 10.1007/s10457-006-9019-4

[B15] Gu L. Dou L. Guo Y. Wang H. Li L. Wang C.. (2019). The WRKY transcription factor GhWRKY27 coordinates the senescence regulatory pathway in upland cotton ( gossypium hirsutum l .). BMC Plant Biol. 19, 1–14. doi: 10.1186/s12870-019-1688-z 30922232 PMC6440019

[B16] Hasanuzzaman M. Bhuyan M. H. M. B. Zulfiqar F. Raza A. Fotopoulos V. (2020). Reactive oxygen species and antioxidant defense in plants under abiotic Stress: revisiting the crucial role of a universal defense regulator. Antioxid. (Basel). 9, 1–52. doi: 10.3390/antiox9080681 PMC7465626 32751256

[B17] Hussain A. Zafar Z. U. Athar H. ur R. (2014). Flooding tolerance in cotton (Gossypium hirsutum l.) at early vegetative and reproductive growth stages. Pakistan J. Bot. 46, 1001–1009.

[B18] Jia W. Ma M. Chen J. Wu S. (2021). Molecular sciences plant morphological, physiological and anatomical adaption to flooding stress and the underlying molecular mechanisms. Int. J. Mol. Sci. 22, 1088. doi: 10.3390/ijms22031088 33499312 PMC7865476

[B19] Kaushal J. Singh S. G. Arya S. K. (2018). Catalase enzyme: application in bioremediation and food industry. Biocatal. Agric. Biotechnol. 16, 192–199. doi: 10.1016/j.bcab.2018.07.035

[B20] Kongdin M. Mahong B. Lee S. K. Shim S. H. Jeon J. S. Ketudat Cairns J. R. (2021). Action of multiple rice β-glucosidases on abscisic acid glucose ester. Int. J. Mol. Sci. 22, 1–14. doi: 10.3390/ijms22147593 PMC8303963 34299210

[B21] Le Provost G. Lesur I. Lalanne C. Da Silva C. Labadie K. Aury J. M.. (2016). Implication of the suberin pathway in adaptation to waterlogging and hypertrophied lenticels formation in pedunculate oak (Quercus robur l.). Tree Physiol. 36, 1330–1342. doi: 10.1093/treephys/tpw056 27358207

[B22] Licausi F. Van Dongen J. T. Giuntoli B. Novi G. Santaniello A. Geigenberger P.. (2010). HRE1 and HRE2, two hypoxia-inducible ethylene response factors, affect anaerobic responses in arabidopsis thaliana. Plant J. 62, 302–315. doi: 10.1111/j.1365-313X.2010.04149.x 20113439

[B23] Loreti E. Valeri M. C. Novi G. Perata P. (2018). Gene regulation and survival under hypoxia requires starch availability and Metabolism. Plant Physiol. 176, 1286–1298. doi: 10.1104/pp.17.01002 29084901 PMC5813553

[B24] Luan H. Guo B. Pan Y. Lv C. Shen H. Xu R. (2018). Morpho-anatomical and physiological responses to waterlogging stress in different barley (Hordeum vulgare L.) genotypes. Plant Growth Regul. 85, 399–409. doi: 10.1007/s10725-018-0401-9

[B25] Mansoor S. Wani O. A. Lone J. K. Manhas S. Kour N. Alam P.. (2022). Reactive oxygen species in Plants: from source to sink. Antioxidants 11, 1–14. doi: 10.3390/antiox11020225 PMC8868209 35204108

[B26] Marczak Ł. Augustyniak A. (2016). Water deficit affects primary metabolism differently in two lolium multiflorum / festuca arundinacea introgression forms with a distinct capacity for photosynthesis and membrane regeneration. Front. Plant Sci. 7. doi: 10.3389/fpls.2016.01063 PMC4958636 27504113

[B27] Najeeb U. Bange M. P. Tan D. K. Y. Atwell B. J. (2015). Consequences of waterlogging in cotton and opportunities for mitigation of yield losses. AoB Plants. 7, 1–18. doi: 10.1093/aobpla/plv080 PMC4565423 26194168

[B28] Nevalainen T. J. (2010). 1-cysteine peroxiredoxin: a dual-function enzyme with peroxidase and acidic Ca2+-independent phospholipase A2 activities. Biochimie 92, 638–644. doi: 10.1016/j.biochi.2010.01.019 20138108

[B29] Ohta A. (2016). A metabolic immune Checkpoint: adenosine in tumor microenvironment. Front Immunol 7, 109. doi: 10.3389/fimmu.2016.00109 27066002 PMC4809887

[B30] Parent C. Berger A. Folzer H. Dat J. Crevècoeur M. Badot P.. (2008). A novel nonsymbiotic hemoglobin from oak: cellular and tissue specificity of gene expression. New Phytol. 177, 142–154. doi: 10.1111/j.1469-8137.2007.02250.x 17986182

[B31] Park S. Lee C. Kim S. Lim Y. Lee H. Nam S.. (2020). Plant physiology and biochemistry selection of flooding stress tolerant sweetpotato cultivars based on biochemical and phenotypic characterization. Plant Physiol. Biochem. 155, 243–251. doi: 10.1016/j.plaphy.2020.07.039 32781274

[B32] Patankar H. V. Al-Harrasi I. Al Kharusi L. Jana G. A. Al-Yahyai R. Sunkar R.. (2019). Overexpression of a metallothionein 2A gene from date palm confers abiotic stress tolerance to yeast and arabidopsis thaliana. Int. J. Mol. Sci. 20, 1–21. doi: 10.3390/ijms20122871 PMC6627811 31212812

[B33] Perata P. Dongen J. T. (2011). Rule pathway for protein destabilization. Nature 479, 419–422. doi: 10.1038/nature10536 22020282

[B34] Qi X. Li Q. Ma X. Qian C. Wang H. Ren N.. (2019). Waterlogging-induced adventitious root formation in cucumber is regulated by ethylene and auxin through reactive oxygen species signalling. Plant Cell Environ. 42, 1458–1470. doi: 10.1111/pce.13504 30556134

[B35] Qian L. Chen X. Wang X. Huang S. Luo Y. (2020). The effects of flood, drought, and flood followed by drought on yield in cotton. Agronomy 10, 1–18. doi: 10.3390/agronomy10040555

[B36] Rajhi I. Yamauchi T. Takahashi H. Nishiuchi S. Shiono K. (2011). Identification of genes expressed in maize root cortical cells during lysigenous aerenchyma formation using laser microdissection and microarray analyses. New Phytol. 190, 351–368. doi: 10.1111/j.1469-8137.2010.03535.x 21091694

[B37] Sadras V. O. (1995). Field crops research review compensatory growth in cotton after loss of reproductive organs. F. Crop Res. 40, 1–18. doi: 10.1016/0378-4290(94)00088-T

[B38] Sharma A. Shahzad B. Rehman A. Bhardwaj R. Landi M. (2019). Response of phenylpropanoid pathway and the role of polyphenols in plants under abiotic stress. Molecules 24, 1–22. doi: 10.3390/molecules24132452 PMC6651195 31277395

[B39] Shimamura S. Yamamoto R. Nakamura T. Shimada S. Komatsu S. (2010). Stem hypertrophic lenticels and secondary aerenchyma enable oxygen transport to roots of soybean in flooded soil. Ann. Bot. 106, 277–284. doi: 10.1093/aob/mcq123 20660468 PMC2908175

[B40] Song L. Valliyodan B. Prince S. Wan J. Nguyen H. T. (2018). Characterization of the XTH gene family: new insight to the roles in soybean flooding tolerance. Int. J. Mol. Sci. 19, 1–20. doi: 10.3390/ijms19092705 PMC6164600 30208612

[B41] Springer B. Werr W. Starlinger P. Bennett D. C. Zokoliea M. Freeling M. (1986). The shrunken gene on chromosome 9 of zea mays l is expressed in various plant tissues and encodes an anaerobic protein. Mol. Gen. Genet. 4, 461–468. doi: 10.1007/BF00338083 2436026

[B42] Takahama U. Oniki T. Shimokawa H. (1996). A possible mechanism for the oxidation of sinapyl alcohol by peroxidase- dependent reactions in the Apoplast: enhancement of the oxidation by hydroxycinnamic acids and components of the apoplast. Kitakyushu, Japan: Plant Cell Physiology, Kyushu Dental College, Vol. 37. 499–504. doi: 10.1093/oxfordjournals.pcp.a028972

[B43] Taylor-kearney L. J. Flashman E. (2021). Targeting plant cysteine oxidase activity for improved submergence tolerance. Plant J. 109, 1–10. doi: 10.1111/tpj.15605 34817108

[B44] Tian L. Bi W. Liu X. Sun L. Li J. (2019). Effects of waterlogging stress on the physiological response and grain-filling characteristics of spring maize (Zea mays l.) under field conditions. Acta Physiol. Plant 41, 1–14. doi: 10.1007/s11738-019-2859-0

[B45] Wang G. Li Q. Dzakpasu M. Gao X. Yuwen C. Wang X. C. (2018a). Impacts of different biochar types on hydrogen production promotion during fermentative co-digestion of food wastes and dewatered sewage sludge. Waste Manage. 80, 73–80. doi: 10.1016/j.wasman.2018.08.042 30455029

[B46] Wang M. Meng D. Zhang P. Wang X. Brennan C. Li S.. (2018b). Antioxidant protection of nobiletin , 5-demethylnobiletin , tangeretin , and 5-demethyltangeretin from citrus peel in saccharomyces cerevisiae. J. Agric. Food Chem. 66, 3155–3160. doi: 10.1021/acs.jafc.8b00509 29526093

[B47] Wang T. Wang Y. Liu X. Gao X. Hu K. (2022). Combined transcriptomics and metabolomics analyses in grass carp under anesthetic stress. Front. Cell. Infect. Microbiol. 12. doi: 10.3389/fcimb.2022.931696 PMC9309352 35899048

[B48] Xu Q. T. Yang L. Zhou Z. Q. Mei F. Z. Qu L. H. Zhou G. S. (2013). Process of aerenchyma formation and reactive oxygen species induced by waterlogging in wheat seminal roots. Planta 238, 969–982. doi: 10.1007/s00425-013-1947-4 23975011

[B49] Yin D. Chen S. Chen F. Guan Z. Fang W. (2010). Morpho-anatomical and physiological responses of two dendranthema species to waterlogging. Environ. Exp. Bot. 68, 122–130. doi: 10.1016/j.envexpbot.2009.11.008

[B50] Yoon Y. Seo D. H. Shin H. Kim H. J. Kim C. M. Jang G. (2020). The role of stress-responsive transcription factors in modulating abiotic stress tolerance in plants. Agronomy 10, 1–23. doi: 10.3390/agronomy10060788

[B51] Yu F. Tan Z. Fang T. Tang K. Liang K. Qiu F. (2020). A comprehensive transcriptomics analysis reveals long non-coding RNA to be involved in the key metabolic pathway in response to waterlogging stress in maize. Genes (Basel). 11, 1–19. doi: 10.3390/genes11030267 PMC7140884 32121334

[B52] Zahra R. Id N. Id S. S. Z. Mukhtar M. S. Amin I. Mishra B.. (2019). Transcriptomic analysis of cultivated cotton gossypium hirsutum provides insights into host responses upon whitefly-mediated transmission of cotton leaf curl disease. PloS One 14, 1–21. doi: 10.1371/journal.pone.0210011 PMC6366760 30730891

[B53] Zeng R. Chen T. Wang X. Cao J. Li X. Xu X.. (2021). Physiological and expressional regulation on photosynthesis , starch and sucrose metabolism response to waterlogging stress in peanut. Front. Plant Sci. 12. doi: 10.3389/fpls.2021.601771 PMC8283264 34276712

[B54] Zhang C. Chen J. Huang W. Song X. Niu J. (2021). Transcriptomics and metabolomics reveal purine and phenylpropanoid metabolism response to drought stress in dendrobium sinense , an endemic orchid species in hainan island. Front. Genet. 12. doi: 10.3389/fgene.2021.692702 PMC8283770 34276795

[B55] Zhang Y. Chen Y. Lu H. Kong X. Dai J. Li Z.. (2016). Growth, lint yield and changes in physiological attributes of cotton under temporal waterlogging. F. Crop Res. 194, 83–93. doi: 10.1016/j.fcr.2016.05.006

[B56] Zhang Y. Kong X. Dai J. Luo Z. Li Z. Lu H.. (2017). Global gene expression in cotton (Gossypium hirsutum l.) leaves to waterlogging stress. PloS One 12, 1–24. doi: 10.1371/journal.pone.0185075 PMC5617174 28953908

[B57] Zhang H. Li G. Yan C. Zhang X. Cao N. Le M.. (2022). Elucidating the molecular responses to waterlogging stress in cucumis melo by comparative transcriptome profiling. Horticulturae 8, 891. doi: 10.3390/horticulturae8100891

[B58] Zhang Y. Liu G. Dong H. Li C. (2020). ScienceDirect waterlogging stress in cotton: damage, adaptability, alleviation strategies, and mechanisms. Crop J. 2, 1–14. doi: 10.1016/j.cj.2020.08.005

[B59] Zhao N. Li C. Yan Y. Cao W. Song A. Wang H.. (2018). Comparative transcriptome analysis of waterlogging-sensitive and waterlogging-tolerant chrysanthemum morifolium cultivars under waterlogging stress and reoxygenation conditions. Int. J. Mol. Sci. 19, 1–21. doi: 10.3390/ijms19051455 PMC5983694 29757964

