# Supplementary material for: Transcriptomic and metabolomic analyses reveal the potential mechanism of waterlogging resistance in cotton (Gossypium hirsutum L.)
Source: Front Plant Sci. 2023 Jun 12;14:1088537. doi: 10.3389/fpls.2023.1088537 (PMC10319419; doi:10.3389/fpls.2023.1088537)
Supplement: Supplementary file 1 [file Table_1.docx]

**Table S1.** Summary of sequences analysis in Cotton roots.

| **Sample** | **Number of raw reads** | **Number of Clean reads** | **Q20 (%)** | **Q30 (%)** | **GC content (%)** |
| --- | --- | --- | --- | --- | --- |
| CJ56T01 | 47,028,750 | 46,642,720 | 98.23 | 94.44 | 43.8 |
| CJ56T02 | 44,767,350 | 44,376,308 | 98.4 | 94.73 | 43.69 |
| CJ56T03 | 43,610,008 | 42,939,420 | 97.31 | 92.35 | 43.83 |
| CJ56T101 | 448,564,46 | 44,526,712 | 98.07 | 94.06 | 44.43 |
| CJ56T102 | 41,210,366 | 40,922,972 | 98.07 | 93.99 | 44.14 |
| CJ56T103 | 48,970,516 | 48,512,604 | 97.6 | 93.3 | 44.38 |
| CJ56T201 | 41,395,810 | 41,051,166 | 97.56 | 93.23 | 44.62 |
| CJ56T202 | 46,037,090 | 45,689,526 | 97.54 | 93.18 | 44.95 |
| CJ56T203 | 44,926,162 | 44,570,976 | 97.53 | 93.06 | 45.29 |
| CJ72T01 | 41,622,556 | 41,207,260 | 98.18 | 94.26 | 43.41 |
| CJ72T02 | 51,333,142 | 50,822,448 | 98.17 | 94.25 | 43.58 |
| CJ72T03 | 42,132,012 | 41,549,866 | 97.4 | 92.49 | 43.75 |
| CJ72T101 | 46,007,666 | 45,582,636 | 98.23 | 94.47 | 44.03 |
| CJ72T102 | 44,863,542 | 44,430,090 | 98.28 | 94.57 | 44.36 |
| CJ72T103 | 42,812,192 | 42,480,000 | 97.16 | 92.07 | 44.27 |
| CJ72T201 | 47,255,378 | 46,848,066 | 97.49 | 93.09 | 44.36 |
| CJ72T202 | 43,544,774 | 43,173,502 | 97.41 | 92.97 | 45.19 |
| CJ72T203 | 45,210,920 | 44,843,076 | 97.52 | 93.16 | 44.07 |

Q20: The percentage of bases with a Phred value >20

Q30: The percentage of bases with a Phred value >30

GC GC content

**Table S2.** Gene ontology classifications of 43,894 orthologous genes under waterlogging stress.

| **GO ID** | **GO terms/description** | **Number of genes** |
| --- | --- | --- |
|  | **Biological process** |  |
| GO:0022610 | biological adhesion | 6 |
| GO:0065007 | biological regulation | 4107 |
| GO:0071840 | cellular component organization or biogenesis | 856 |
| GO:0009987 | cellular process | 15675 |
| GO:0032502 | developmental process | 74 |
| GO:0040007 | growth | 37 |
| GO:0002376 | immune system process | 7 |
| GO:0051179 | localization | 3391 |
| GO:0008152 | metabolic process | 17783 |
| GO:0051704 | multi−organism process | 187 |
| GO:0032501 | multicellular organismal process | 236 |
| GO:0048519 | negative regulation of biological process | 95 |
| GO:0048518 | positive regulation of biological process | 104 |
| GO:0050789 | regulation of biological process | 4016 |
| GO:0000003 | reproduction | 8 |
| GO:0022414 | reproductive process | 163 |
| GO:0050896 | response to stimulus | 2289 |
| GO:0023052 | signaling | 956 |
| GO:0044699 | single−organism process | 10731 |
|  | **Cellular component** |  |
| GO:0005623 | cell | 4972 |
| GO:0044464 | cell part | 4972 |
| GO:0005576 | extracellular region | 100 |
| GO:0032991 | macromolecular complex | 2496 |
| GO:0016020 | Membrane | 2918 |
| GO:0044425 | membrane part | 2817 |
| GO:0031974 | membrane−enclosed lumen | 204 |
| GO:0043226 | Organelle | 3445 |
| GO:0044422 | organelle part | 964 |
|  | **Molecular function** |  |
| GO:0016209 | antioxidant activity | 318 |
| GO:0005488 | Binding | 25575 |
| GO:0003824 | catalytic activity | 17034 |
| GO:0016530 | metallochaperone activity | 4 |
| GO:0098772 | molecular function regulator | 537 |
| GO:0060089 | molecular transducer activity | 163 |
| GO:0001071 | nucleic acid binding transcription factor activity | 1465 |
| GO:0005198 | structural molecule activity | 1006 |
| GO:0000988 | transcription factor activity, protein binding | 96 |
| GO:0005215 | transporter activity | 1437 |

**Table S3.** Transcriptome gene pathway analysis. Total gene ratio involved in those pathways, total up and down DEGs corresponding to the pathways and their respective P-value in cotton roots of CJ1831056 and CJ1831072**.**

| **VARIETY** | **Gene pathways** | **Gene ratio** | | **Up DEGs** | | **Down DEGs** | | **P-value** | |
| --- | --- | --- | --- | --- | --- | --- | --- | --- | --- |
|  |  | **56T10 VS56T0** | **56T20 VS56T0** | **56T10 VS56T0** | **56T20 VS56T0** | **56T10 VS56T0** | **56T20 VS56T0** | **56T10 VS56T0** | **56T20 VS56T0** |
| CJ1831056 | Starch and sucrose metabolism | 151/3890 | 171/4815 | 60 | 64 | 91 | 107 | 3.30E-05 | 0.001483 |
|  | Phenylpropanoid biosynthesis | 139/3890 | 218/4815 | 20 | 20 | 119 | 198 | 0.009004 | 2.26E-13 |
|  | Glycolysis / Gluconeogenesis | 136/3890 | 171/4815 | 61 | 64 | 75 | 107 | 1.03E-05 | 2.93E-08 |
|  | Cysteine and methionine metabolism | 119/3890 | 129/4815 | 41 | 39 | 78 | 90 | 9.97E-06 | 0.001994 |
|  | Pyruvate metabolism | 104/3890 | 133/4815 | 34 | 43 | 70 | 90 | 0.001299 | 1.87E-05 |
| CJ1831072 | Phenylpropanoid biosynthesis | 104/1421 | ND | 13 | ND | 91 | 91 | 5.53E-18 | ND |
|  | Glycolysis /Gluconeogenesis | 79/1421 | 126/2591 | 40 | 54 | 39 | 39 | 3.90E-12 | 4.21E-15 |
|  | Starch and sucrose metabolism | 60/1421 | ND | 24 | ND | 36 | 36 | 0.00313 | ND |
|  | Cysteine and methionine metabolism | 54/1421 | 83/2591 | 18 | 31 | 36 | 52 | 4.13E-05 | 9.43E-05 |
|  | Pyruvate metabolism | 52/1421 | 92/2591 | 17 | 30 | 35 | 62 | 2.48E-05 | 3.92E-08 |
|  | Fatty acid metabolism | 52/1421 | 69/2591 | 17 | 21 | 35 | 48 | 2.48E-05 | 1.09E-05 |
|  | Purine metabolism | ND | 68/2591 | ND | 36 | ND | 32 | ND | 0.010546 |

**Table S4.** Differentially expressed XTH (xyloglucan endotransglucosylase/hydrolase protein) in two cotton genotype under waterlogging.

| **Gene ID** | **CJ56T10vs56T0** | **CJ56T20vs56T0** | **Gene Description** | **CJ72T10vs72T0** | **CJ72T10vs72T0** |
| --- | --- | --- | --- | --- | --- |
|  | **Log2FC** | **Log2FC** |  | **Log2FC** | **Log2FC** |
| LOC107930656 | 1.4 | 2.1 | Probable xyloglucan endotransglucosylase/hydrolase protein XTH-30 | ND | 1.3 |
| LOC107938513 | −1.7 | −2.2 | Probable xyloglucan galactosyltransferase AtGT17 | −1.3 | −2.3 |
| LOC107926340 | 2.8 | 3.5 | Xyloglucan endotransglucosylase/hydrolase protein XTH-22 | 2.3 | 3.5 |
| LOC107959252 | 3.0 | 2.2 | Xyloglucan endotransglucosylase/hydrolase protein XTH9 | 2.6 | 3.5 |

**Table S5.** Top 10 metabolites pathways. (Pathway names, total metabolites involved in those pathways, metabolites significantly accumulated in present study (hits), impact (The influential metabolite factors obtained) and false discovery rate (FDR) in cotton roots of CJ1831056 and CJ1831072 identified by Pathway Analysis using Arabidopsis thaliana as the pathway library

| **Stress time** | **Metabolite Pathway** | **Total**  **Number of metabolites** | **Compounds**  **Hit** | **Impact** | **P-value** | **FDR** | **Metabolite type** |
| --- | --- | --- | --- | --- | --- | --- | --- |
| Zero stress | Biosynthesis of unsaturated fatty acids | 42 | Eicosapentaenoic acid | 0 | 2.36 | 1 | NEG |
| 10 Days | Purine metabolism | 61 | Hypoxanthine | 1 | 0.15 | 1 | NEG |
|  | Phenylpropanoid biosynthesis | 45 | Sinapyl alcohol | 0.02562 | 1.39 | 1 | POS |
| 20 Days | Valine, leucine and isoleucine biosynthesis | 26 | L-Valine | 0.0178 | 1.00 | 1 | POS |
|  | Galactose metabolism | 26 | Melibiose | 0.07042 | 1.00 | 1 | POS |
|  | Alanine, aspartate and glutamate metabolism | 22 | L-Glutamic acid | 0.33333 | 1.3162 | 1 | NEG |
|  | Citrate cycle (TCA cycle) | 20 | Citric acid | 0.0952 | 1.3987 | 1 | NEG |
|  | Amino sugar and nucleotide sugar metabolism | 41 | Glucose 1-phosphate | 0.11022 | 0.81287 | 1 | NEG |
|  | Arginine and proline metabolism | 38 | L-Glutamic acid | 0.14004 | 0.87032 | 1 | NEG |
|  | Starch and sucrose metabolism | 30 | Glucose 1-phosphate | 0.17167 | 1.0567 | 1 | NEG |

**Table S6.** The primers used for qRT-PCR

| **Gene symbol** | **Gene ID** | **Forward Primer sequences (5‘-3’)** | **Reverse Primer sequences (3‘-5’)** |
| --- | --- | --- | --- |
| XTH9 | LOC107959252 | GAC AGC AGG CGA TGA GCA TCC | AGC ATT GGT CCA GTT GGT CTT CAC |
| MT1 | LOC107936947 | CTG CGG TGA CAA CTG CTC GTA AG | ATC CCA TCC GTT TCG TTC AAG ATC G |
| RBOHD | LOC107957571 | GGT GGT TTG TGA AGG GAG TGG AAG | GGA GTG GCT AGT GTG AAG GCA ATA G |
| PER1 | LOC107916248 | CCA GCA ACC ACA GGG CGA AAC | GGG CAC CGA TGG CGA AAT CAC |
| ADH | LOC107910431 | TTG GGT TGT TTG GCT ATT GCT TTG C | GAC TTC ATC CTT CTG CGG TGG TG |
| RAP2-3 | LOC107896651 | GGA AAT GGG CGG CTG AGA TAA GAG | GCT TGG CGG CTT CAT CGT AGG |
