# Supplementary figures and images for: Transcriptomic and metabolomic analyses reveal the potential mechanism of waterlogging resistance in cotton (Gossypium hirsutum L.)
Source: Front Plant Sci. 2023 Jun 12;14:1088537. doi: 10.3389/fpls.2023.1088537 (PMC10319419; doi:10.3389/fpls.2023.1088537)

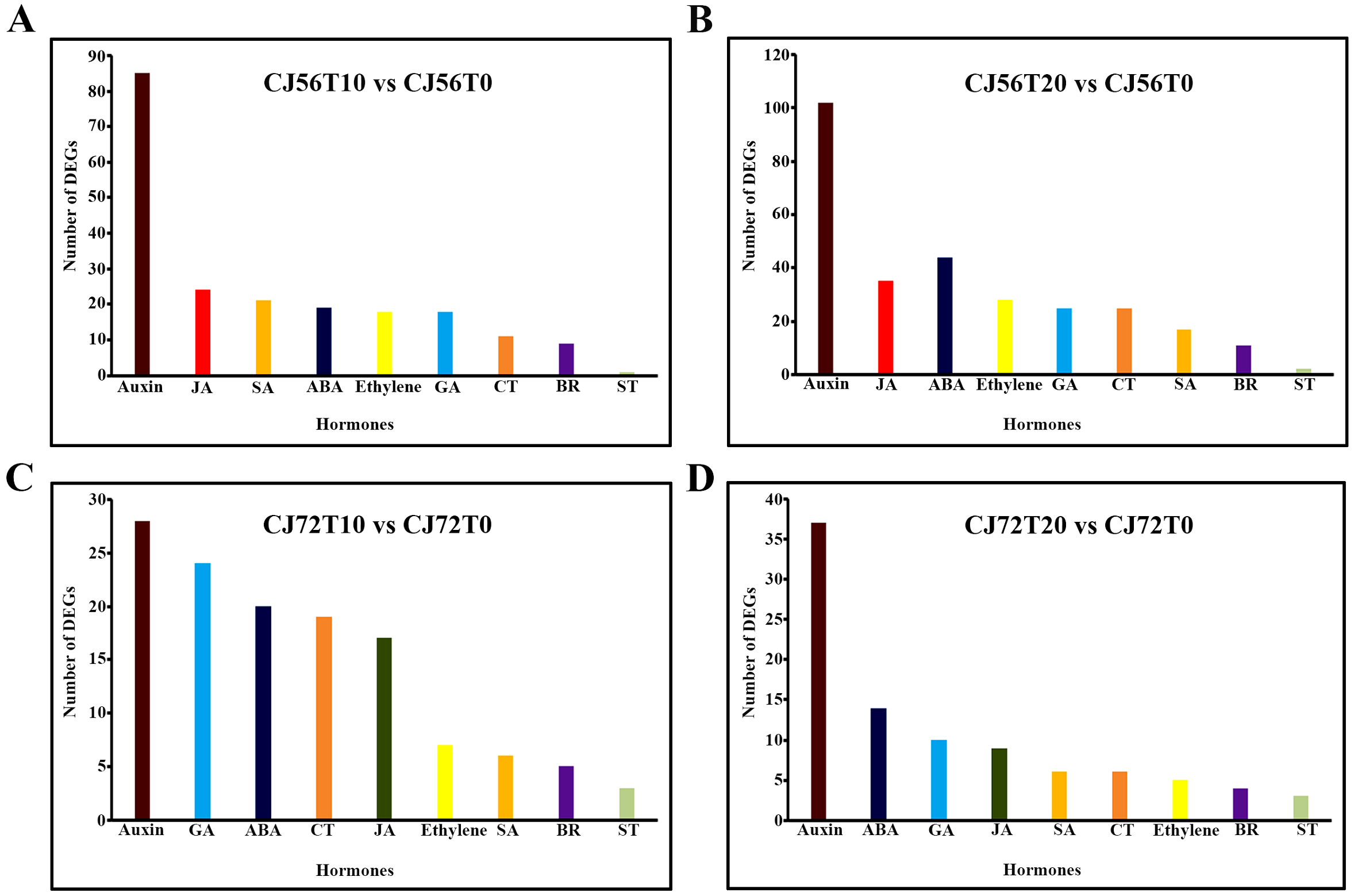

Supplement: Supplementary Figure 1 — Number of hormone DEGs in cotton roots during waterlogging stress. (A) DEGs in CJ56T10vsCJ56T0. (B) DEGs in CJ56T20vsCJ56T0. (C) DEGs in CJ72T10vsCJ72T0. (D) DEGs in CJ72T20vsCJ72T0. [file Image_1.tif]

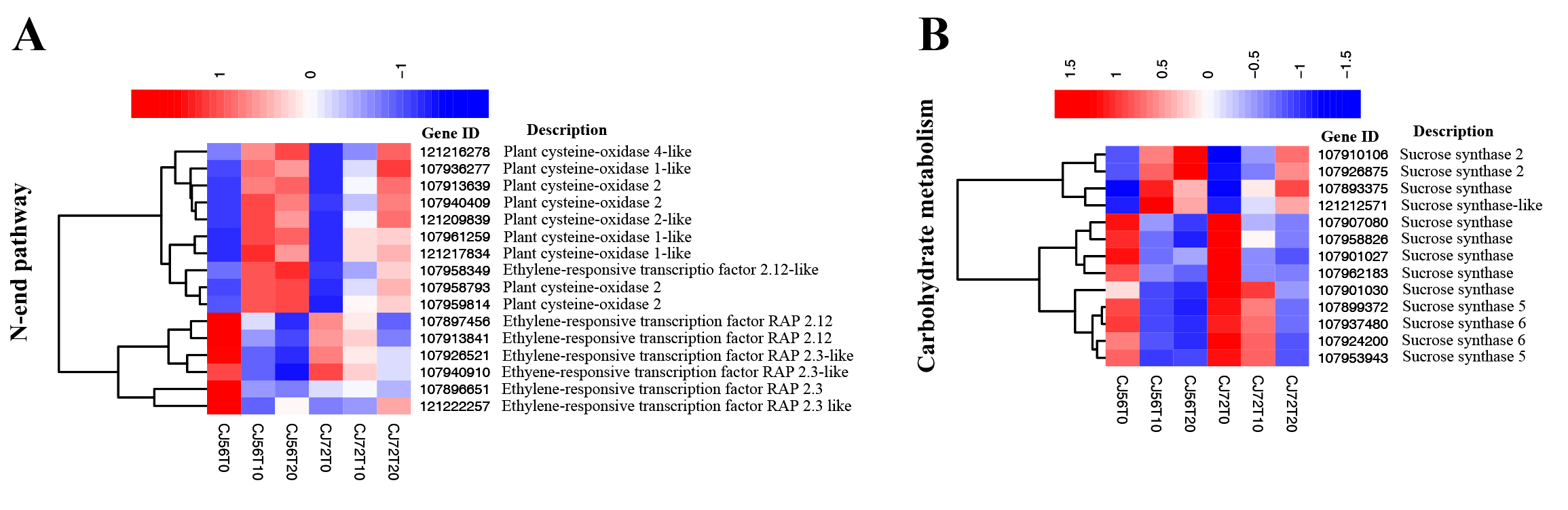

Supplement: Supplementary Figure 2 — Heat map clustering analysis of DEGs. (A) Clustering analysis of DEGs involved in the N-end rule pathway. (B) Clustering analysis of DEGs involved in carbohydrate metabolism. The bar represents the scale of the expression levels of each gene (log2 FPKM) in each sample, as indicated by red/blue rectangles. Red rectangles represent the high expression of genes, and blue rectangles represent the low expression. [file Image_2.tif]

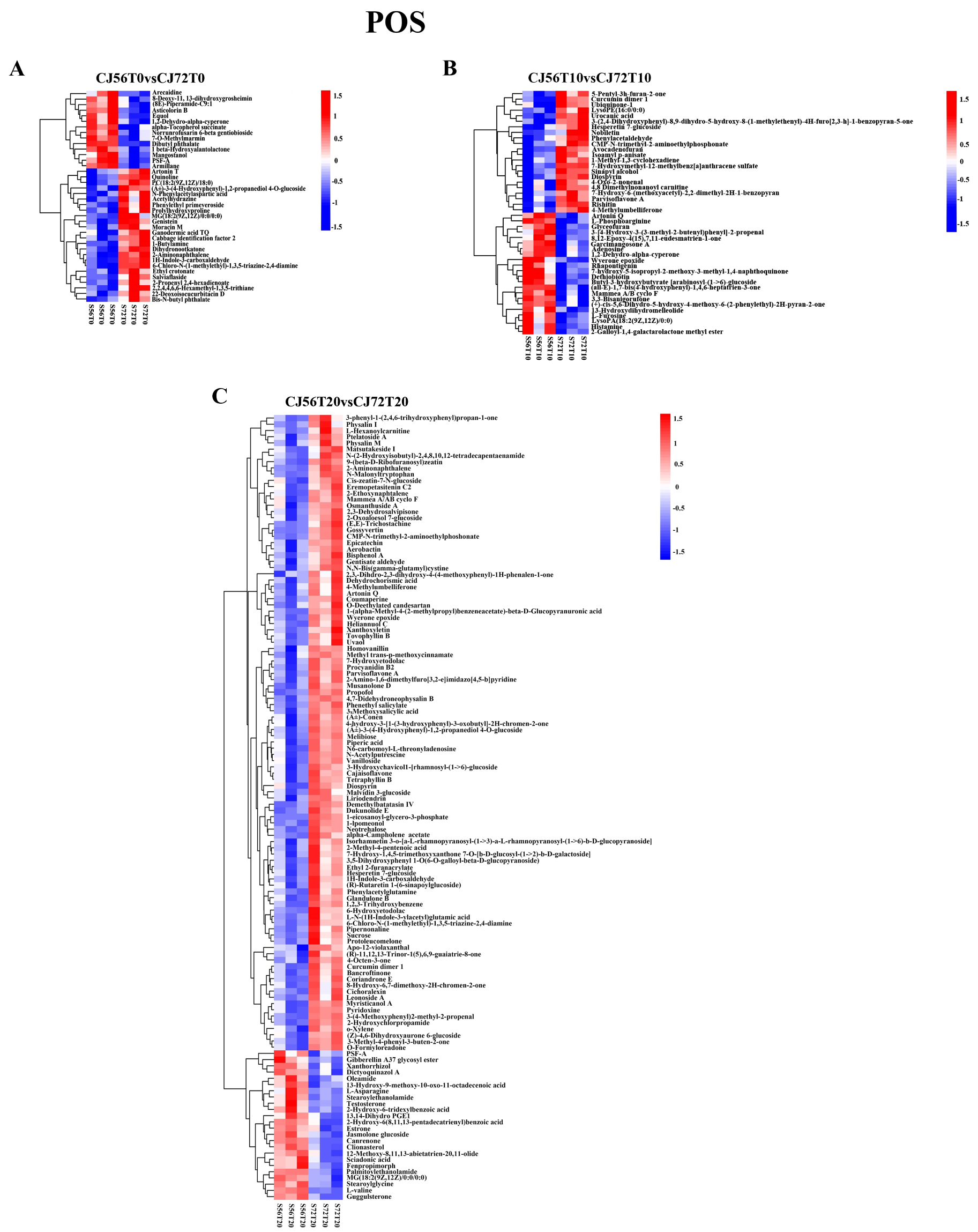

Supplement: Supplementary Figure 3 — Analysis of accumulated metabolites at 10 days of waterlogging in cotton waterlogged roots. (A) Metabolites absolute value of the fold change. (B) The bubble plot represents a metabolic pathway. (C) Hierarchical cluster analysis. The abscissa represents the different experimental groups, the ordinate represents the comparative metabolites of the group, and the color blocks at different positions represent the relative expression amount of the metabolites at the corresponding positions. Red indicates high expression of the substance, and blue indicates low expression. [file Image_3.tif]

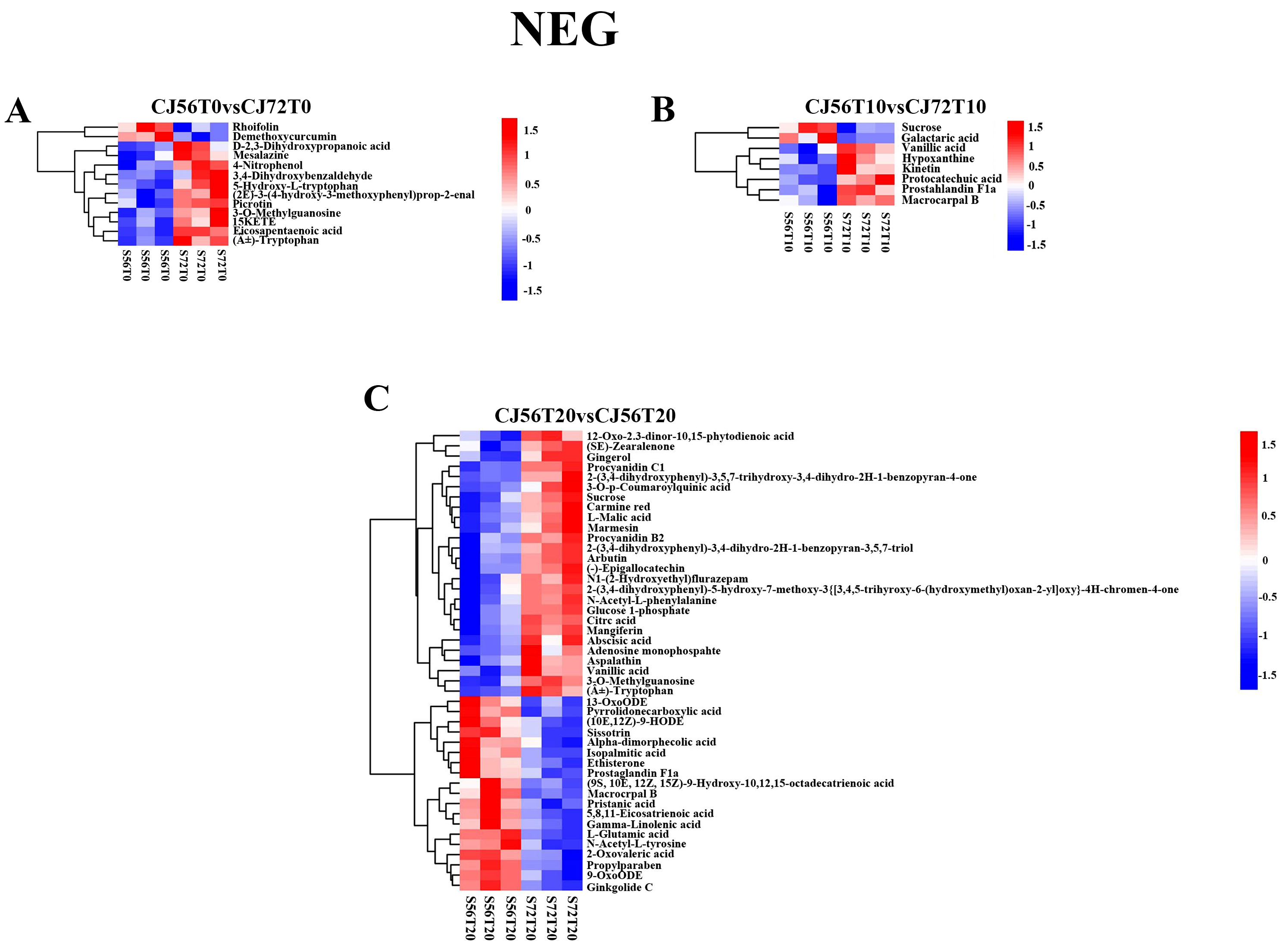

Supplement: Supplementary Figure 4 — Analysis of accumulated metabolites at 20 days of waterlogging in cotton waterlogged roots. (A) Metabolites’ absolute value of the fold change (waterlogging/Control) at 20 days in cotton roots. (B) The bubble plot represents a metabolic pathway. (C) Hierarchical cluster analysis. The abscissa represents the different experimental groups, the ordinate represents the comparative metabolites of the group, and the color blocks at different positions represent the relative expression amount of the metabolites at the corresponding positions. Red indicates high expression of the substance, and blue indicates low expression. [file Image_4.tif]
